# Photocatalytic Performance of Undoped and Al-Doped ZnO Nanoparticles in the Degradation of Rhodamine B under UV-Visible Light:The Role of Defects and Morphology

**DOI:** 10.3390/ijms232415459

**Published:** 2022-12-07

**Authors:** Alessandra Piras, Chiara Olla, Gunter Reekmans, An-Sofie Kelchtermans, Dries De Sloovere, Ken Elen, Carlo Maria Carbonaro, Luca Fusaro, Peter Adriaensens, An Hardy, Carmela Aprile, Marlies K. Van Bael

**Affiliations:** 1 Laboratory of Applied Materials Chemistry, Unit of Nanomaterials Chemistry, Chemistry Department, University of Namur, NISM, Rue de Bruxelles, 61, 5000 Namur, Belgium; 2 DESINe Group, Institute for Materials Research (imo-imomec), Hasselt University, Agoralaan Building D, 3590 Diepenbeek, Belgium; 3 Department of Physics, University of Cagliari, Cittadella Universitaria, I-09042 Monserrato, Italy; 4 Analytical and Circular Chemistry (ACC), Institute for Materials Research (imo-imomec), Hasselt University, Agoralaan Building D, 3590 Diepenbeek, Belgium; 5 EnergyVille, Thor Park 8320, 3600 Genk, Belgium; 6 Imec, Division Imomec, Wetenschapspark 1, 3590 Diepenbeek, Belgium

**Keywords:** nanomaterials, ZnO, Al-doped ZnO, Rhodamine B, photocatalysis, green light-irradiation, photoluminescence, solid-state ^27^Al-NMR

## Abstract

Quasi-spherical undoped ZnO and Al-doped ZnO nanoparticles with different aluminum content, ranging from 0.5 to 5 at% of Al with respect to Zn, were synthesized. These nanoparticles were evaluated as photocatalysts in the photodegradation of the Rhodamine B (RhB) dye aqueous solution under UV-visible light irradiation. The undoped ZnO nanopowder annealed at 400 °C resulted in the highest degradation efficiency of ca. 81% after 4 h under green light irradiation (525 nm), in the presence of 5 mg of catalyst. The samples were characterized using ICP-OES, PXRD, TEM, FT-IR, ^27^Al-MAS NMR, UV-Vis and steady-state PL. The effect of Al-doping on the phase structure, shape and particle size was also investigated. Additional information arose from the annealed nanomaterials under dynamic N_2_ at different temperatures (400 and 550 °C). The position of aluminum in the ZnO lattice was identified by means of ^27^Al-MAS NMR. FT-IR gave further information about the type of tetrahedral sites occupied by aluminum. Photoluminescence showed that the insertion of dopant increases the oxygen vacancies reducing the peroxide-like species responsible for photocatalysis. The annealing temperature helps increase the number of red-emitting centers up to 400 °C, while at 550 °C, the photocatalytic performance drops due to the aggregation tendency.

## 1. Introduction

Based on the world water development report 2020 [1], among the most critical contemporary global issues, the conservation of water resources associated with global climate change is of high environmental importance. Water resources are currently contaminated with various organic, inorganic and microbial pollutants. Organic contaminants, which are mainly employed in processing products such as fabrics, cosmetics, leather, plastic, ceramics, paper, ink-jet printing, pharmaceuticals, etc. [2,3,4], are due to the discharge of organic dyes through industrial wastewater. In aquatic life, different pollutants were identified, including textile dyes, surfactants, insecticides, pesticides and heavy metals [5]. Exposure to dyes at a small level of less than 1 mg L^−1^ can seriously affect the water quality of the aquatic environment [3]. In fact, Rhodamine B (RhB), Methyl Orange (MO) and Methylene Blue (MB) are commonly used mutagenic, toxic and non-biodegradable dyes hazardous to aquatic life. These dyes also threaten human lives due to their high potential of being carcinogenic [6]. In particular, traces of organic dyes in water can result in ailments such as abdominal disorders, irritations, anemia and many more [7,8].

In the textile industry, up to 200,000 tons of dyes are dispersed in water bodies every year during the dyeing and finishing manipulations due to the inefficiency of the dyeing process [9]. Therefore, the removal of pollutant dyes from wastewater is needed. Among the conventional treatments available nowadays, various chemical and physical processes such as chemical precipitation and separation, adsorption and coagulation methods are in use [10]. However, these methods lead to incomplete dye degradation and only transfer the contaminant from one phase to another [11]. Advanced Oxidation Processes (AOPs), based on semiconducting materials, have emerged in recent years as an alternative to conventional methods [12]. Indeed, through these processes, reactive species such as hydroxyl radicals can be generated and used as active species to oxidize the organic contaminants quickly and non-selectively. Heterogeneous photocatalysis utilizing oxide-based nanomaterials is of particular interest owing to its ability to destroy water-soluble organic pollutants in water and wastewater [13].

Ideally, the system design concept for the photocatalytic degradation reaction includes the use of particles suspended in water as photocatalysts irradiated by light. In this process, the photocatalyst plays a fundamental role. The utilized materials are semiconductors characterized by an electronic band structure, where the highest occupied energy band, called the valence band (V.B.), and the lowest empty band, the conduction band (C.B.), are separated by an energy bandgap. The absorption of photons with equivalent to or higher energies than the semiconductor bandgap leads to the generation of the electron (e^−^)–hole (h^+^) pairs in the semiconductor particles. A charge separation follows due to the migration of these photogenerated carriers in the semiconductor particles. Then, the surface chemical reactions between these carriers with various compounds (e.g., H_2_O, hydroxyl radicals) occur. Electrons and holes may also recombine with each other without participating in any chemical reactions. The oxidizing agents attack the organic pollutants present on or near the catalytic surface until there is complete mineralization into harmless species [14,15].

Until now, ZnO-based nanomaterials have been studied as heterogeneous photocatalysts for dye degradation because they are abundant, environmentally friendly, non-toxic, insoluble in neutral water and economical [16]. However, the efficiency of zinc oxide as a photocatalyst is still limited due to the rapid recombination of electron–hole pairs and the lower activity in the visible region than in the UV-range [17]. Doping of ZnO is an interesting way to optimize and tune its optical, electrical, magnetic and structural performance. Zinc oxide can be observed in three polymorphs: wurtzite, zinc blend and rock salt [18]. Under ambient conditions, the thermodynamically stable phase is the wurtzite structure. Structurally, wurtzite has a hexagonal closed-packed arrangement of O^2-^ anions where half of the tetrahedral sites are occupied by the Zn^2+^ cations. The other half of the tetrahedral sites and all the octahedral sites are empty. These latter sites procure possible dopant sites. n-type and p-type doped ZnO materials have been extensively studied because they exhibit interesting properties for industrial applications [18,19]. Undoped ZnO displays n-type conductivity, traditionally attributed to intrinsic defects such as zinc excess at the interstitial positions and the lack of oxygen. The n-type conductivity can also be acquired by doping with post-transition metal ions such as Al [20], Ga [21] and In [22]. It is known that the trend in doping efficiency is related to the size of the trivalent dopant (In > Ga > Al) with respect to the Zn^2+^ ion [21]. The greater similarity between the dopant and the host cation size allows a more favorable lattice substitution. Indeed, the ionic radii for Zn^2+^, In^3+^, Ga^3+^ and Al^3+^ in CN = 4 coordination are 0.60, 0.62, 0.47 and 0.39 Å, respectively [21]. These dopants mentioned above have fully occupied d orbitals, hence no possible internal d-d transitions. However, they stimulate the formation of native defects in the ZnO lattice (for instance, zinc interstitials and oxygen vacancies). Such defects generate mid-bandgap energy levels which are reported to increase carrier trapping leading to rapid non-radiative recombination of the electron–hole pairs within the semiconducting materials. Although doping can be used to enhance the general efficiency of the photocatalyst, many contributions to a changing performance have to be considered, such as preparation method [23], particle morphology [12,24], surface properties [25,26] and defects [27], dopant concentration [2] as well as the charge transfer dynamics of the discoloration process [28].

In this work, aluminum was selected as a dopant for the ZnO semiconductor material because of its abundancy, low price and suitability. Several articles have been published regarding the conductivity enhanced through n-type doping when Al is incorporated into the zinc oxide crystal lattice [19,20,21,29]. Some authors [30] also noticed that the electronic properties of the doped oxide are influenced by the crystallographic position of the aluminum dopant in the ZnO lattice. Indeed, the Al^3+^ ion can occupy the empty tetrahedral sites, and also substitute a Zn^2+^ ion in the tetrahedral geometry, to furnish a free electron (charge carrier) which improves the conductivity. Likewise, an interstitial octahedral coordinated site can be occupied by the Al^3+^ ion. It is reported that when Al^3+^ ions occupy the octahedral site, the conductivity decreases [30,31]. Thus, the Al^3+^ dopant should be placed in a substitutional, tetrahedral position to optimize the ZnO host material’s electronic properties.

The insertion of post-transition metals as dopants creates defects responsible for enhanced functionalities and might lead to enhanced charge separation [32]. Therefore, we synthesized, characterized and investigated undoped and Al-doped ZnO nanoparticles with increasing percentages of aluminum with respect to Zn for photocatalytic degradation under UV-visible light irradiation of Rhodamine B. Although many authors have reported on the photodegradation of Rhodamine B by ZnO [7,33,34,35], it is difficult, if not impossible, to directly compare their photocatalytic performances due to the many variables (e.g., reaction conditions, light source, reactor setup, etc.) that are not standardized. We also examined the effect of the thermal treatment in a dynamic nitrogen atmosphere at 400 °C and 550 °C on the catalysts, focusing on the role played by the defects.

## 2. Results and Discussion

Designing a photocatalyst requires attention to the composition (e.g., Al/Zn ratio) and the morphology. Nanosized dimensions are crucial for a material to function as an efficient photocatalyst due to a large number of atoms at the surface with their distinct optical, crystallographic and electronic properties. Thus, the strategy consisted in synthesizing undoped and Al-doped ZnO particles in the nanoscale to achieve large surface areas with consequent benefits for its performance. To this end, a method previously described by Momot et al. [19] was followed, where the authors presented an applicable solvothermal route to nanocrystalline Al-doped ZnO based on the reaction between zinc (II) acetylacetonate and aluminum (III) acetylacetonate as precursors and benzylamine used as solvent and reactant, Figure S1. The dopant concentrations range from 0 at% to 5 at% with respect to Zn. To investigate and evaluate any possible effects of the aluminum dopant on the zinc oxide structure, Inductively Coupled Plasma Optical Emission Spectroscopy (ICP-OES), Powder X-ray Diffraction (PXRD), Transmission Electron Microscopy (TEM), Fourier Transform Infrared spectroscopy (FT-IR), Solid-State ^27^Al-Magic Angle Spinning Nuclear Magnetic Resonance (SS ^27^Al-MAS NMR), UltraViolet-Visible spectroscopy (UV-vis) and steady-state Photoluminescence spectroscopy (PL) have been used. The thermal treatment under nitrogen atmosphere was applied to investigate its effect on the position of the dopant and the related performance change. In particular, the annealing in a dynamic nitrogen atmosphere was performed on the ZnO (ZO) and Al-doped ZnO (AZO-05 and AZO-5) at 400 and 550 °C. Moreover, commercial ZnO (CZO) is used as reference material. The resulting synthesized and commercial samples were coded as listed in Table 1.

**Figure 1:**
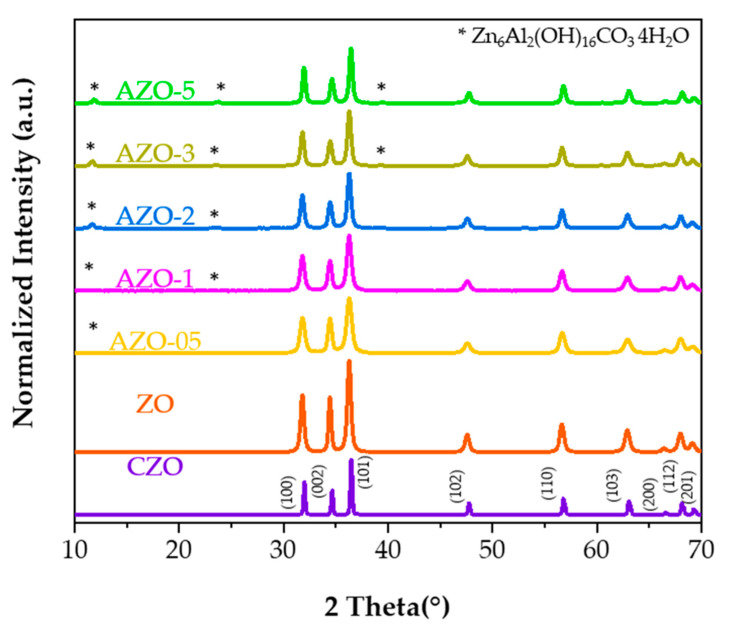
X-ray diffraction patterns of undoped ZnO and Al-doped ZnO powders as-synthesized with different aluminum content. CZO is used as reference material. Symbol: *—corresponds to the secondary phase: Zn_6_Al_2_(OH)_16_CO_3_ 4H_2_O.

The powder XRD patterns of commercial ZnO, undoped ZnO and Al-doped ZnO materials displayed in Figure 1, where the CZO was used as reference solid.

All patterns match the hexagonal wurtzite crystal phase (PDF card 89-1397) [10]. The characteristic diffraction peaks at 2θ = 31.79°, 34.44°, 36.28°, 47.59°, 56.61°, 62.91°, 66.41°, 68.04° and 69.17° indicate the reflection from (100), (002), (101), (102), (110), (103), (200), (112) and (201) crystal planes, respectively. At an increasing amount of aluminum inserted into the zinc oxide lattice, an additional crystalline phase, identified as Zn_6_Al_2_(OH)_16_CO_3_∙4H_2_O (PDF card 38-0486), is observed. Although the Al^3+^ ion is smaller than the Zn^2+^ ion, its incorporation is complex and incomplete [36]. Therefore, depending on slight variations of the experimental conditions [30,37], a segregated phase of aluminum external to the zinc oxide lattice might explain the presence of different diffraction peaks in the XRD patterns [20,38,39].

The doping efficiency also depends on the crystallographic position in the ZnO lattice and is majorly correlated to the post-treatments and synthesis method [30].

A quantitative determination of dopant incorporation within the ZnO structure was evaluated using ICP-OES, Table S1. Although the presence of a secondary phase (vide infra), might lead to an incorrect composition analysis, the results display a good relationship between the nominal amounts of aluminum added during the synthesis mixture and the actual aluminum found in the final solid. The missing aluminum content most probably was removed by the repeated washing procedures. This would suggest that doping ZnO quasi-spherical nanoparticles with aluminum is more efficient at lower reaction molar ratios. Similar observations for the experimental aluminum content are made in the literature [30,40].

**Figure 2:**
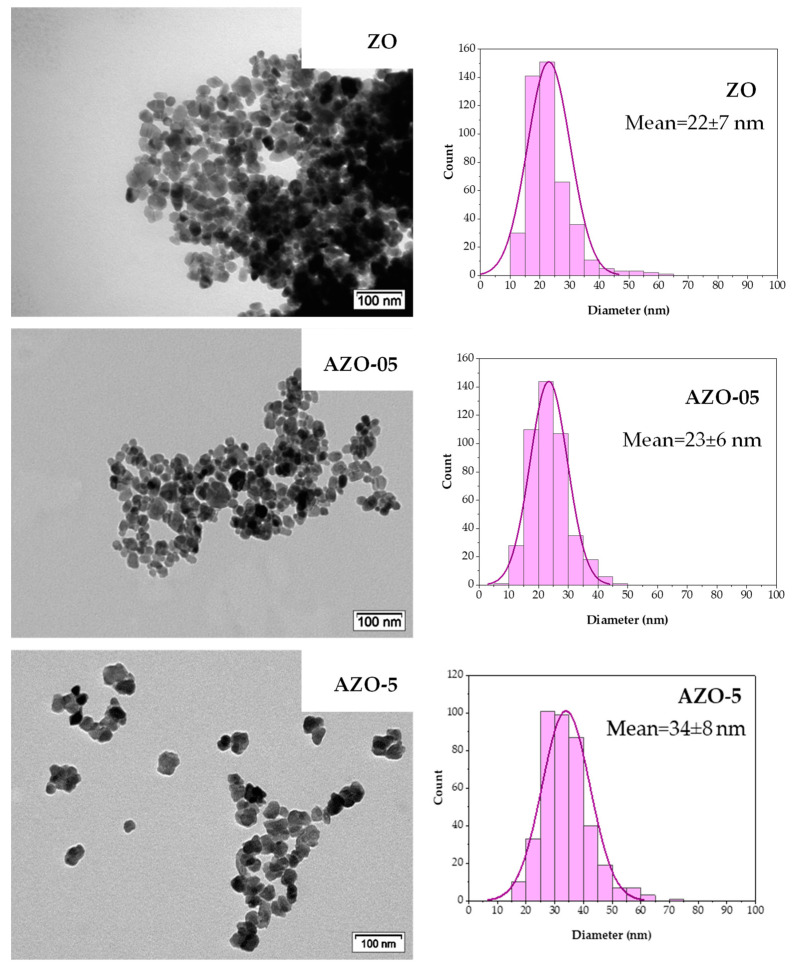
TEM micrographs of the ZO, AZO-05 and AZO-5 as-synthesized materials supported with the corresponding particle size histograms.

TEM was used to evaluate the morphology and size of the nanoparticles. As displayed in Figure 2, the synthesis produced quasi-spherical nanoparticles exhibiting diameters ranging between 10 and 70 nm for ZO and AZO, highlighting the success of the synthesis method. From TEM investigation, the nominal aluminum content in the AZO nanoparticles as-synthesized does not affect the average diameter and the size distribution of the quasi-spheres when the aluminum content is low. In contrast, an increase in diameter is observed for the AZO-5 NPs. It was previously reported [41,42,43,44,45] that the morphology of the particles is not affected at low aluminum content while it can be strongly altered (or modified) for increased aluminum content. For instance, Kelchtermans and coworkers [46] demonstrated that at an aluminum concentration of 10 at%, the presence of both Al-doped ZnO nanoparticles and nanorods can be detected. Pinna et al. [47] observed a mixture of spheres and rods for pure ZnO, and the presence of different morphologies was particularly evident when impurities were present. Generally, when the spherical morphology is maintained, a decrease in the diameter of the AZO nanoparticles is reported.

A further structural investigation was performed after annealing the ZO and AZO nanopowders in a nitrogen atmosphere at 400 and 550 °C; see Figures S2 and S3.

The particle size distribution histograms show that thermal treatment affects the zinc oxide NPs shape and size. The particles size goes from 22 ± 7 nm for the bare ZO, to 42 ± 21 nm for the same material annealed at 400 °C and up to 51 ± 21 nm for the nanoparticles annealed at 550 °C. Furthermore, the aggregation tendency is more pronounced by annealing at 550 °C temperature. A broader particle size distribution together with the appearance of particles of different shape was already observed in the samples annealed at 400 °C. Indeed, the particle size distribution presents a wider standard deviation for the annealed materials due to the variety of morphologies displayed.

**Figure 3:**
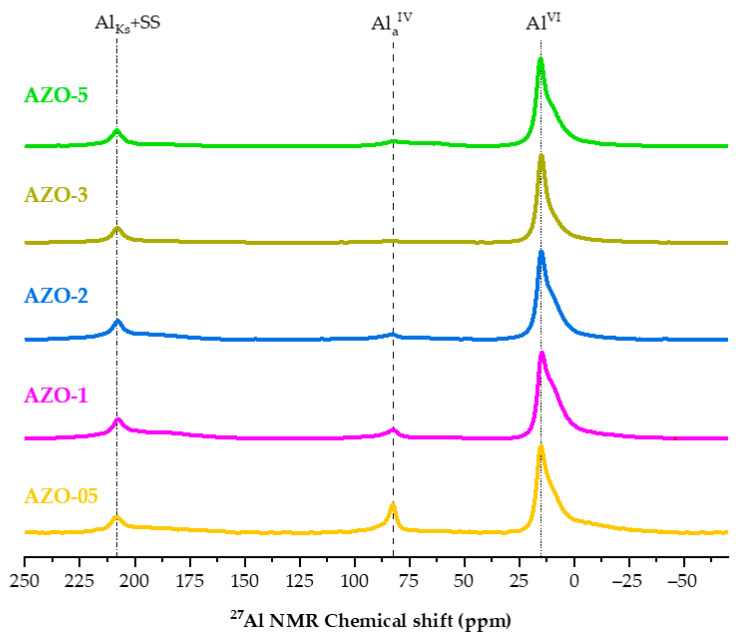
^27^Al MAS NMR of AZO quasi−spherical nanoparticles as−prepared with a different nominal aluminum content. The lines highlight the peaks assigned to the 6−fold coordinated aluminum, Al^VI^, 4−fold coordinated aluminum, Al_a_^IV^ and a spinning−side band (SS) of the octahedral signal also containing a Knight−shift signal, Al_Ks_ + SS.

Solid-state ^27^Al MAS NMR can provide information on the local environment of the aluminum ions inserted into the zinc oxide structure [30,48]. Therefore, the technique is used to study the position of aluminum in the crystalline lattice [19,30,48]. Figure 3 presents the solid-state ^27^Al MAS NMR spectra of the AZO as-synthesized series, in which the signal assignments can be made based on the correlation between the observed chemical shift value and the coordination number of the aluminum atoms.

Four principal signals are often observed in the AZO ^27^Al MAS NMR spectra: the first signal around 10 ppm results from a 6-fold coordination, i.e., Al coordinated to 6 oxygen atoms (octahedral, Al^VI^) [29]. The peak often has a narrow linewidth indicating a highly ordered, crystalline environment, implying that the aluminum is incorporated into the crystalline ZnO lattice during the synthesis. In this case, however, the general line shape of the octahedral signal is broadened by superposition with a broad contribution originating from a portion of the aluminum atoms in an amorphous, disordered environment [48]. ^27^Al nucleus is quadrupolar and can interact with an external magnetic field as well as with an electric field gradient generated in the surrounding environment. Therefore, in a symmetrical environment the generated signal is sharp, while in an asymmetrical environment, the signal shape is distorted and broadened [49]. The second signal around 82 ppm is referred to as the 4-fold coordination, i.e., Al coordinated to 4 oxygen atoms (tetrahedral, Al_a_^IV^). In agreement with results reported in the literature [30], higher aluminum contents imply a decreasing fraction of aluminum occupying tetrahedral sites. The third signal around 200 ppm is (partly) due to a spinning-side band (SS) of the octahedral signal but can also contain a Knight-shift signal (Al_Ks_). In the literature, some authors [50] reported the observation of a Knight-shift signal in their NMR spectra. The mechanism of appearance of this signal in metals has been explained [51], while the exact origin of this signal in ZnO samples has been suggested [19]. These three signals are all observed in the spectra of the AZO samples in this study. Sometimes, a fourth, broad signal appears around 70 ppm indicating the presence of aluminum in a 5-fold coordination state (Al^V^) or in a distorted 4-fold coordination (Al_b_^IV^) [29]. This signal is clearly observed in the spectra of the AZOs after annealing in N_2_ at 400 and 550 °C (Figure S5).

Based on the characterization results obtained by PXRD, ICP-OES and ^27^Al MAS NMR, the discussion presented below will focus mainly on three selected materials, ZO, AZO-05 and AZO-5. Indeed, the bare ZnO, AZO-05 and AZO-5 are representative for the entire AZO series.

It is reported in literature [19] that an enhanced conductivity of the AZO materials is obtained by annealing in a reductive atmosphere at increased temperatures. The authors showed that aluminum atoms occupying interstitial positions in the zinc oxide lattice can migrate to the substitutional positions, at the same time creating interstitial Zn atoms. They also provided evidence that the origin of the Knight-shift peak observed in their NMR spectra is related to the formed complex of aluminum in these substitutional positions and Zn at interstitial positions. To evaluate how the annealing procedure might affect the photocatalytic activity of the AZO semiconductors, the AZO-05 and AZO-5 samples were annealed in a tube furnace under a dynamic nitrogen atmosphere at temperatures of 400 °C and 550 °C. Figure S5 shows the ^27^Al MAS NMR spectra of these annealed AZO samples. Compared to the spectra of the not-annealed samples shown in Figure 3, the spectra of the annealed AZOs show a much broader signal for the octahedral Al^VI^ next to the presence of a broad signal around 70 ppm from 5-fold coordinated (Al^V^) or distorted 4-fold coordinated (Al_b_^IV^) aluminum. It emerges that a significant part of the aluminum atoms migrates from an octahedral position to a tetrahedral, as well as distorted tetrahedral (or pentahedral) position, after annealing. These results are in agreement with the literature [19]. Taking the comparison between AZO-05 and AZO-05-400 as an example, it is clear that thermal annealing favors the migration of Al atoms from octahedral environments towards tetrahedral and distorted 4-fold or 5-fold coordination sites. Moreover, by increasing the annealing temperature from 400 °C to 550 °C, aluminum atoms almost only migrate from 4-fold coordination sites towards the distorted 4-fold or 5-fold coordination sites. This behavior is also observed comparing AZO-05-400 to AZO-5-400. In addition, for the annealed AZO nanoparticles, a lower amount of aluminum doping results in an increase of aluminum in a metallic-state environment (Al_Ks_).

**Figure 4:**
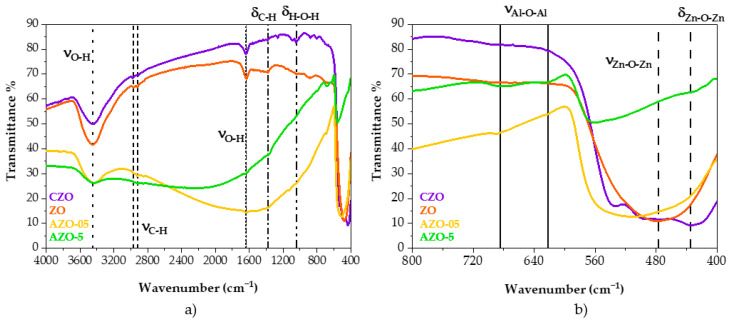
FT-IR spectra of quasi-spherical CZO, ZO, AZO-05 and AZO-5 not-annealed nanoparticles, (**a**). Magnification of the FT-IR fingerprint zone, (**b**).

The ^27^Al-NMR spectra have demonstrated that the aluminum in the zinc oxide lattice is located mainly in tetrahedral and octahedral sites. However, it is still unclear whether the tetrahedral sites arise from substituting the Zn^2+^ ions. It might be possible that the signal of tetrahedral aluminum is due to Al^3+^ ions occupying the vacant interstitial tetrahedral positions instead of substituting Zn^2+^ ions. Therefore, to further assess the effect of aluminum as a dopant, FT-IR measurements were performed. This technique is used to obtain information about the vibrational stretching and bending of the undoped and Al-doped zinc oxide materials in the infrared region. The transmittance of the KBr pellets containing CZO, ZO and AZO materials was measured in the interval of 4000–400 cm^−1^; see Figure 4.

The fingerprint zone (bands between 400–800 cm^−1^) illustrates the stretching and bending modes of Zn-O bonds at ca. 477 and 435 cm^−1^, respectively [33,52]. The bands at ca. 3400, 1637 and 1045 cm^−1^ are related to the stretching and bending modes of O-H groups in physisorbed water [30]. The peaks observed at ca. 2970, 2921, and 1375 cm^−1^ are due to C-H asymmetric stretching and bending vibrations of alkane groups [53,54]. These are ascribed to impurities present in the KBr powder. The emergence of the bands with low intensity at ca. 622 and 685 cm^−1^ in the AZO samples are characteristic of Al-O stretching mode [53,55,56]. Together with the peaks mentioned above, a pronounced band between 800 and 3000 cm^−1^ is observed for both AZO samples, while it is absent for the undoped materials. This band, called the Localized Surface Plasmon Resonance (LSPR) band [19,20,21,30], is a clear signature of n-doping of the AZO nanoparticles caused by the increased free electron density. The position of the LSPR band depends on the density of charge carriers. It is also affected by other parameters, such as nanoparticles size, shape, defects and segregation of dopants [21].

One of the requirements to exhibit the LSPR band is to have the Al^3+^ ions dopant substituting the positions of the Zn^2+^ ions instead of occupying the vacant tetrahedral positions of the wurtzite structure [30]. Therefore, the FT-IR spectra recorded of the powders of the quasi-spherical AZO nanoparticles are indicative of substitutional doping.

A closer look at the fingerprint zone in Figure 4 shows that from ZO to AZO-5, probably due to the insertion of the dopant and consequent displacement of the zinc in the oxide lattice, there is a modification of the peak’s shape and a shift towards higher wavenumbers. This behavior is also observed in the literature for ZnO doped with other metals [52].

Infrared spectra of AZO and ZO nanomaterials, annealed at 400 and 550 °C under nitrogen flow, are reported in Figure S6. A comparison between the band’s shape of the samples in Figure 4 and Figure S6A highlight how this thermal treatment modifies the LSPR. The band of the AZO-05 is less pronounced, while for AZO-5, it is broader and more intense when the samples are annealed at 400 °C, indicating some modification in terms of free charge carriers and their mobility. In the fingerprint zone, increasing the amount of dopant in the nanomaterials leads to a visible modification of the vibrational modes of the Zn-O peaks in terms of intensity and shape. Generally, as shown in the spectra on the right side in Figure 4, the commercial zinc oxide displays a trimodal band due to its multitude of shapes, including rod-like, quasi-spherical and agglomerated particles (see TEM, Figure S4). The ZO quasi-spherical nanoparticles present a monomodal band shape, instead, which is slightly modified for AZO-05 and more different in AZO-5. Therefore, the modification of this band shape and the shift towards a higher wavenumber could be related to the dopant insertion and a variation in morphology. In fact, in Figure S6A,B the role of dopant is principally highlighted, while Figure S6C shows the role of the annealing temperature. The TEM images show that the size of the nanoparticles starts to grow with the temperature and that they also tend to agglomerate into bigger particles, modifying the band’s shape. It can be noted that ZO without annealing presents a monomodal band (fingerprint zone in Figure 4 and Figure S6C), whereas ZO-400 presents a bimodal band which we tentatively ascribe to the changes in morphology (see TEM Figure S2), and ZO-550 is again monomodal, potentially due to the substantial aggregation of the nanoparticles. These observations agree with the literature where spherical ZnO nanoparticles tend to agglomerate to bigger particles due to the calcination temperature [57].

### Photocatalytic Degradation of Rhodamine B Dye

ZnO is one of the most widely used semiconducting materials for the photodegradation of organic pollutants in water [58,59,60]. Even though many scientific publications report the design of novel catalysts, the photocatalytic response of material depends mainly on the particle size distribution, morphology and crystalline phase of the selected material. Hence, the photocatalytic activity of as-synthesized ZO and AZO nanoparticles was estimated under UV-visible light irradiation by determining the photodegradation of Rhodamine B (RhB), used as a dye model compound.

The degradation of the RhB dye was verified via UV-vis analysis of the solution after removal of the solid catalyst (when needed). In the absence of photocatalyst, the degradation of Rhodamine B under UV-visible light irradiation was found negligible, whilst the dye–catalyst suspension did undergo discoloration under UV and green light irradiation.

Table 2 displays the photocatalytic activity in terms of dye degradation (%) of CZO, ZO, AZO-05 and AZO-5 nanomaterials as-synthesized. The activity was monitored after 20 min of reaction under UV-C and UV-A light irradiation. The CZO was tested as reference. Under UV-C light irradiation, RhB degradation occurred in the presence of all the catalysts. These results evidenced that the photocatalytic activity decreases while increasing the amount of aluminum in the catalysts. Among the catalysts containing aluminum, the best photocatalytic performance is shown by the AZO-05 sample. This behavior is not unexpected as the literature reports a similar behavior for Al-doped and other ZnO-based systems [61,62]. Indeed, it was reported that increasing the aluminum content of doped samples results in higher photocatalytic activity for the Al/Zn molar ratio up to 1.0%. Higher aluminum contents provoked a reduction of photocatalytic efficiency. The same considerations can be applied to the photocatalytic activities obtained under UV-A light irradiation. However, among all the catalysts synthesized in our work, the best performance has been shown by the ZO material.

Table 3 displays the RhB degradation expressed in percentage for a one-hour reaction under green light irradiation for the ZO, AZO-05, and AZO-5 nanomaterials and a four-hour reaction under green light irradiation for the ZO catalyst before and after annealing at 400 and 550 °C. Interestingly, under green light irradiation the best catalytic performance is shown by undoped ZnO solid, of which the activity overpasses that of commercial ZnO. Figure S7 shows the absorbance spectra plotted as a function of the green light exposure time for the RhB samples without the catalyst and with the catalyst that presented the best photocatalytic performance of the synthesized materials. Figure S8 displays the behavior of RhB in the absence of a catalyst, which shows photolysis effects since the dye is sensitive to visible light irradiation [63]. Therefore, the 20% degradation of CZO, shown in Table 3, could be ascribed to the thermal decomposition of the dye as a consequence of the continuous irradiation during one hour with high power lamp and the dark adsorption experiment (ca. 15%, see Figure S9). Whereas when the photocatalytic degradation of the RhB dye occurs under green light irradiation, the amount of dopant present in the zinc oxide lattice seems not to have any positive influence on the photocatalytic activity itself.

The data reported in Table 3 demonstrate that the annealing temperature of the (A)ZO affects the results of the photocatalytic activity. The best result is shown by the undoped ZnO material annealed at 400 °C for 10 min, presenting a photocatalytic activity value of 50% after a one-hour reaction and 81% after four hours of reaction under green light irradiation. However, when the annealing temperature reaches 550 °C, the photocatalytic activity drops, making the catalyst inactive for the same reaction.

The differences between the two annealed ZnO materials can be related to morphological and structural features.

The unexpected increase of the photocatalytic performances under the green light source of our synthesized ZnO nanoparticles (both as-synthesized and annealed at 400 °C) compared to the commercial ZnO bulk sample can be explained by the presence of different types of defects that allow the absorption of light at longer wavelengths (infra gap). Thus, this promotes the degradation process of Rhodamine B dye with less energetic radiation. This is of interest for applications where, instead of UV-light, solar light is used for the photocatalytic degradation.

To explore more in-depth and seek a hypothesis for the photocatalytic behavior of the CZO, ZO and AZO materials, the presence of these defects has been investigated using steady-state PL spectroscopy, which allows a higher sensitivity over optical absorption techniques.

UV-vis reflectance measurements of our samples cannot provide clear information on the specific characteristics of our synthesized particles. Despite the slightly different trends, all spectra show the typical ZnO pattern which is confirmed by the bandgap calculation performed by applying the Schuster–Kubelka–Munk formula [64,65,66,67] and fitting the data with the Boltzmann function according to the methodology proposed by Zanatta [68] (details in the Supplementary Figures S13 and S14 and Table S3). This analysis displays only a small variation in the absorption spectra of the samples, the band gap being estimated in the 378–388 nm range as compared to the theoretical values of 368 nm.

**Figure 5:**
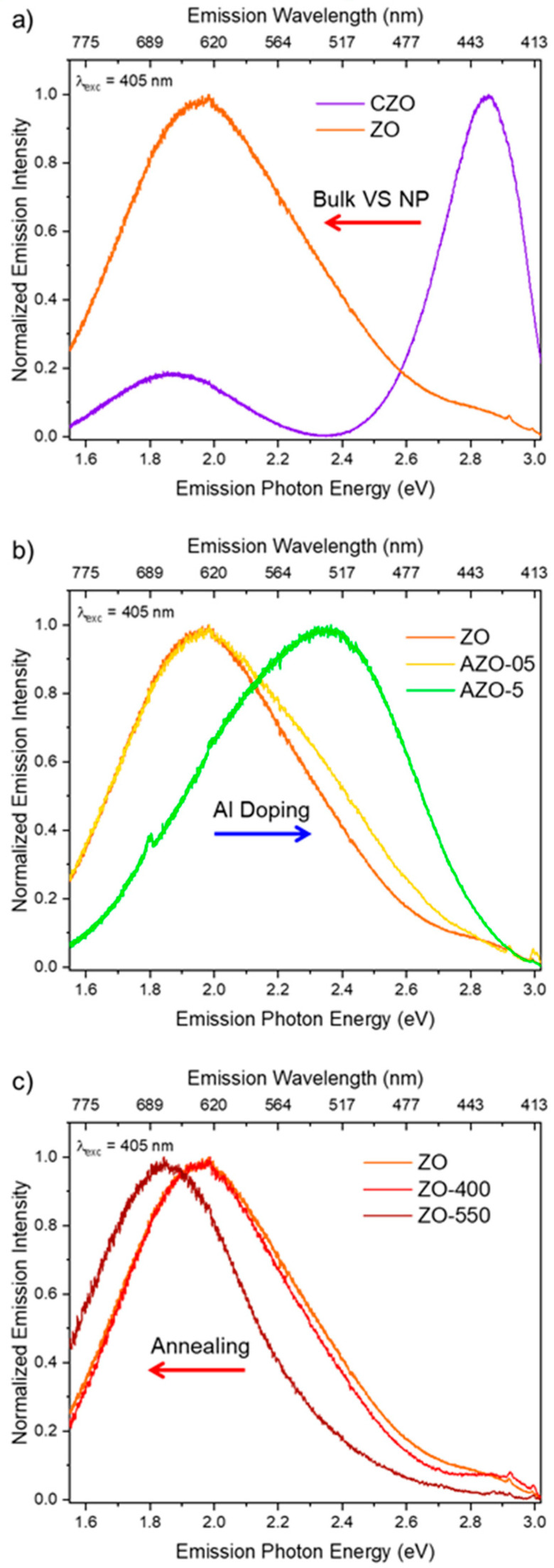
Normalized PL spectra of all samples excited at 405 nm. Comparisons among commercial (bulk) and ZnO nanoparticles (**a**), different Al-doping percentages (**b**), and annealing temperatures (**c**) are displayed.

To bypass the high intrinsic contribution to the emission of ZnO excitons that can cover possible emission signals due to defects and excite only the latter, steady-state PL measurements were carried out in the infra-gap visible region employing 405 and 532 nm laser beam excitations (Figure 5 and Figure S10).

Under 405 nm excitation (Figure 5), the investigated samples show different optical behavior. Figure 5a compares the normalized emission features of the commercial sample made by bulk ZnO (CZO) and the nanosized structure that we have synthesized (ZO). The main emission center in CZO is in the violet–blue region with a secondary weaker peak in the red wavelength range. At the same time, the ZO signal consists of a broad asymmetric red band extending up to the green region with a small contribution in the violet one. This difference in the emission spectra calls for a significant presence of diverse defects within the nanosized ZO responsible for infra-gap absorption and emission in the visible range. Those red-emitting defects could be involved in the observed photocatalytic process through green excitation if they absorb in the same region (vide infra). To further investigate the emission properties of nanosized ZO, we compare in Figure 5b the spectra of ZO and AZO samples. AZO-05 displays a broad emission very similar to the ZO sample, with a slight increase in the relative contribution of the green emission. The latter is further increased in the AZO-5 sample up to being the main emission band. This blue shift caused by aluminum doping could explain its deleterious effect on photocatalysis performances since the relative contribution of red-emitting defects is decreased with respect to the green ones. The opposite trend was displayed after the annealing of the sample in nitrogen (Figure 5c): at 400 °C (ZO-400), there is a slight variation of the luminescence band compared to pristine ZO, while it is red-shifted down to 675 nm after the treatment at 550 °C (ZO-550).

The reported scenario was confirmed under 532 nm excitation (Figure S10), of which the energy is comparable to the one of our photocatalytic processes: ZO nanoparticles present the same red contribution previously observed, with the AZO-5 and ZO-550 samples still slightly blue- and red-shifted, respectively, as compared to the others. These spectra demonstrate the capability of our materials to absorb infra-gap green light, suggesting that the same red-emitting defects could be responsible of our photocatalytic performances in the same energy range. Exciting the CZO sample with the green laser source recorded the absence of any red photoluminescence, supporting the observed poor photocatalytic performances. This is also an indication that the weak red emission displayed in CZO under 405 nm excitation light has a different origin as compared to our synthesized samples.

**Figure 6:**
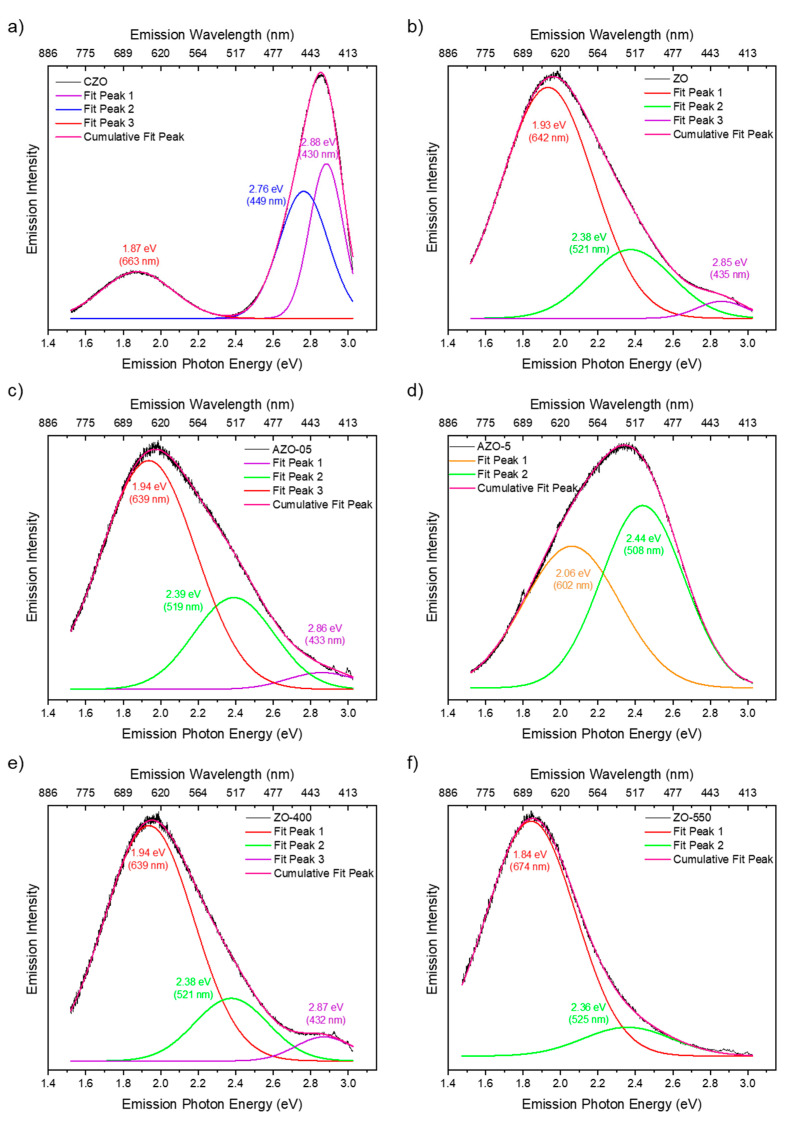
Gaussian deconvolution of emission spectra obtained under 405 nm excitation wavelength of CZO (**a**), ZO (**b**), AZO-05 (**c**), AZO-5 (**d**), ZO-400 (**e**) and ZO-550 (**f**).

To better identify the relative contribution of the different emitting centers, a gaussian deconvolution of the spectra excited at 405 nm was performed (Figure 6).

All the spectra can be fitted assuming two or three contributions in the orange/red, green/blue, and violet range, as reported in Table S2. The analysis confirmed that the red emission band of CZO, centered at about 660 nm, differs from the one detected in the other samples, where the emission band peaked at about 640 nm. This red band is the primary emission band in the ZO, even after annealing at 400 °C (the relative content was about 75% of the overall emission in the undoped sample, 77% after annealing). When annealed at 550 °C, the band is still present but red-shifted by about 35 nm. It should be noted that, different morphologies of the materials may affect scattering and absorption in PL measurements [69]. Therefore, this effect could be related to a change in the environment of the emitting centers, caused by aggregation of the nanoparticles (vide infra); indeed, the TEM images in Figures S2 and S3 show the formation of larger nanoparticle agglomerates. Two other bands, in the green and violet spectral range (at about 520 and 430 nm, respectively) with decreasing relative content, were also added to complete the deconvolutions. Interestingly, the violet band in all samples (except for AZO-5 and ZO-550) peaked at the same spectral position as in the CZO sample, where a further blue band at about 450 nm is required to fit the emission spectrum, instead of the green one of our synthesized samples. Moreover, by doping the samples with a small content of aluminum and through the annealing at 400 °C, these peaks’ positions remain almost unmodified, while increasing the aluminum content largely modified the spectrum, shifting the red band to about 600 nm and largely decreasing the relative contribution of this band (47%) with respect to the green one (53%). According to the detailed study by Zhang et al. [70] on ZnO nanostructures using PL and EPR (Electron Paramagnetic Resonance spectroscopy) measurements, the blue–violet peak of the CZO can be attributed to interstitial Zn in the surface and bulk position, respectively. The surface interstitial Zn band and the related defect contribution are displayed in all samples (the only exception is AZO-5) with a much weaker relative intensity that passes from 35% in CZO to 3–5% in the others. Moreover, the opposite trend of the green and red band relative intensity is worth noting. In the undoped nanoparticles, the green band represents 22% of the area, which increases to 26% in the AZO-05 sample and 53% in the AZO-5. The same authors attributed this green band to O_2_ adsorbed on the surface or charged oxygen vacancies V_o_^+^ (not excluding V_o_ and V_o_^++^) while the broad red one around 2.0 eV might be due to the presence of peroxide-like species O_2_^2−^ interacting with the ZnO surface. Considering these attributions, we propose to explain the photocatalytic properties of the synthesized samples as follows. As stated before, the presence of red-emitting centers promotes the photocatalytic process of our synthesized samples under green light since they can absorb in the green infra-gap region in opposition with commercial ZnO. When ZO is doped with aluminum below 0.5%, tetrahedral positions are taken. At least part of these are aluminum ions substituting zinc ions, as corroborated by the appearance of an LSPR band in FT-IR (see Figure 4a). An increase in the aluminum content up to 5 at% causes the migration of the aluminum atoms mainly towards an octahedral environment, as indicated by solid-state NMR analysis (see Figure 3). Together with the appearance of the aluminum in octahedral environment, XRD analysis (see Figure 1) shows the existence of a secondary phase, which makes us believe that this secondary phase contains an octahedral aluminum environment as well.

Doping with (small amounts of) aluminum in the substitutional Zn^2+^ positions creates oxygen vacancies in the ZnO structure. Along with this, Zn ions are displaced from their lattice position. Hence, the secondary phase, observed in XRD, is probably formed by these displaced Zn^2+^ ions together with the surplus (octahedral) aluminum ions and oxygen. Upon higher aluminum doping, the amount of the secondary phase increases. This may cause more oxygen vacancies to be formed in the ZnO, leading to the increase of the green emission band and, consequently, to the decrease of the photocatalytic performance.

As for the annealed samples, the emission is marginally affected by the thermal treatment at 400 °C whilst its photocatalytic efficiency is increased, from 39 to 50%. The opposite effect is displayed in the ZO-550 sample, where besides the observed red-shift, a decrease in the photocatalytic performances from 39 to 21% is also observed. The explanation of these phenomena is challenging because just a little variation in the mainly red emission spectra that we had associated with the presence of oxidative species was observed. The thermal treatment did cause a change in morphology and distribution of the particles (see Figure S2), and a variation in the aggregation of the nanoparticles, the latter being larger in ZO-550, possibly reducing the photocatalytic surface area.

## 3. Materials and Methods

### 3.1. Synthesis

All chemical reagents were used as received. Zinc acetylacetonate hydrate ((Zn(acac)_2_ · *x*H_2_O) 99.995% m/m% purity, Aldrich), aluminum acetylacetonate (Al(acac)_3_ 99.999% m/m% purity, Aldrich) and benzylamine for synthesis (99% purity, ethanol absolute, for analysis, Merck).

The syntheses of undoped and Al-doped ZnO materials were carried out with 1 g of Zn(acac)_2_ for pure ZnO and in combination with 0.0057g of Al(acac)_3_, ranging from 0.5 to 5 mol% for the AZO nanoparticles. To obtain undoped and Al-doped ZnO nanoparticles by a solvolysis reaction in a reflux setup, Zn(acac)_2_ hydrate was mixed with *x* mol of Al(acac)_3_ and 80 mL of benzylamine and heated to the boiling point (nominal temperature 185 °C). After reaching the boiling point, the mixture was refluxed for four hours under stirring. After cooling to room temperature, the mixture was centrifuged to precipitate the nanoparticles. The particles were then washed three times with ethanol and twice with water. After washing, the obtained powders were dried overnight in the oven at 60 °C. Subsequently, the catalysts were annealed under dynamic nitrogen for 10 min in a tubular oven at 400 and 550 °C.

### 3.2. Characterization Techniques

The Al/Zn ratio in the powders was determined by inductively-coupled plasma optical emission spectrometry (ICP-OES, Perkin Elmer Optima 3300 DV simultaneous spectrometer, PerkinElmer, Waltham, MA, USA). To analyze the Zn and Al content, a small portion of the powdered AZO sample was dissolved in a 5% aqueous nitric acid solution (HNO_3_, 69.0–70.0%, J.T.Baker, for trace metal analysis). The AZO aqueous stock solutions and 1000 ppm Zn, Al standards (Merck) were diluted by 5% HNO_3_ to 1–10 ppm and 10, 5, 2 and 1 ppm concentrations, respectively, for ICP-OES measurements. All ICP analyses were carried out twice. The measurement error was evaluated based on calibration certificates and from statistical analysis of repeated measurements. The following errors were considered: volumetric operations (volumetric flasks, measuring cylinders) and the error of concentrations/purity of commercial chemicals. For calculations, calibration certificates or information sheets from the manufacturer were used. Powder X-ray Diffraction (PXRD) patterns were collected on a Bruker AXS D8 Discover diffractometer (Cu Kα radiation, LynxEye detector). The undoped and Al-doped ZnO powders were scanned between 2θ = 10° and 70° with a 2θ scan step size of 0.020°. Metal sample holders were used as a support for all powder samples. The profile analysis of the related diffraction patterns was carried out with the program DIFFRAC.EVA (general profile and structure analysis software for powder diffraction data, Bruker Analytical X-ray Systems). Transmission electron microscopy (TEM) images were recorded on a Philips Tecnai 10 with an acceleration voltage of 100 kV. The samples were prepared by dispersing a small quantity of nanopowders in absolute ethanol, depositing them on a carbon-film coated copper mesh and drying them. The particle size distribution was evaluated over 450 nanoparticles. Fourier transform infrared (FT-IR) spectroscopy (Bruker Vertex 70, 32 scans, scan range 4000–400 cm^−1^, resolution 1 cm^−1^) was performed on pellets containing trace amounts of the investigated materials diluted with KBr. The discoloration process was monitored by means of a UV-vis spectrophotometer Agilent Cary 5000. The measures were carried out in the UV-visible range of 200–800 nm, with a quartz cuvette applying baseline corrections. ^27^Al solid-state MAS NMR spectra were acquired on an Agilent VNMRS DirectDrive 400 MHz spectrometer (9.4 T wide bore magnet) equipped with a T3HX 3.2 mm probe. Magic angle spinning (MAS) was performed at 20 kHz in ceramic rotors of 3.2 mm (22μL). AlCl_3_ was used to calibrate the aluminum chemical shift scale (0 ppm). Acquisition parameters used were: a spectral width of 420 kHz, a 90° pulse length of 2.5 μs, an acquisition time of 10 ms, a recycle delay time of 20 s, a line-broadening of 200 Hz and around 10,000 accumulations. UV-vis solid-state reflectance spectra were collected (applying baseline corrections) using a Jasco V-750 spectrophotometer with a spectral bandwidth of 0.2 nm in the 300–500 nm range. Photoluminescence (PL) measurements were performed in backscattering geometry with a confocal micro-Raman system (SOL Confotec MR750) equipped with a Nikon Eclipse Ni microscope. Samples were excited with 405 and 532 nm laser diodes (IO Match-Box series), and the spectral resolution was 0.6 cm^–1^ (average acquisition time 1 s, the average number of acquisitions 3, sensor temperature −23 °C, objective Olympus 10×, grating with 150 grooves/mm, power excitation 3 mW).

### 3.3. Photocatalytic Assessment

The photocatalytic activity of the catalysts was estimated under UV-visible light irradiation, determining the photodegradation of Rhodamine B (RhB), used as a dye model compound. To perform the photocatalytic tests, two homemade photoreactors were used (Figures S11 and S12).

#### 3.3.1. Photodegradation in the UV Range

Quartz beakers containing 20 mL of the RhB aqueous solution (4 mg L^−1^) along with the photocatalyst (5 mg) were used to carry out the photodegradation experiments. A lamp emitting in the UV (UV-C: Osram Hg lamp, 11 W, dominant wavelength 254 nm; UV-A: Phillips Hg lamp, 11 W, dominant wavelength 368 nm), was axially positioned among the four beakers and kept at 1 cm from the magnetic stirrer plate. The reactor was kept at room temperature (24 ± 3 °C) using a fan placed on the back wall of the reactor. The reaction mixtures were stirred using a magnetic stirrer bar of equal length for 30 min in the dark before irradiation to attain the adsorption–desorption equilibrium of the substrate. The reactions were stopped after 20 min. The suspensions were centrifuged to separate the catalysts from the aqueous dye solution and analysed by UV-vis spectroscopy, considering the main absorption peak of this dye in the visible range, located at 554 nm. The degradation % is calculated using Formula (1):

(1) $$\mathrm{Degradation}\;\%=\frac{\left(A_{0}-A\right)}{A_{0}}*100$$

where *A*_0_ is the absorbance of the starting solution before reaction and *A* is the final absorbance measured after the reaction. Photolysis tests of the RhB dye were carried out under UV light irradiation. The single photocatalytic test was performed four times to ensure the reproducibility of the data.

#### 3.3.2. Photodegradation in the Visible Range

A quartz beaker containing 20 mL of the RhB aqueous solution (4 mg L^−1^) and the photocatalyst (5 mg) were used to carry out the photodegradation tests. A lamp emitting in the visible range (dominant wavelength 525 nm, 18 W Evoluchem LED) was axially positioned and kept at 10 cm from the top of the beaker. To avoid the contribution of the UV-A light, a 405 nm long-pass edge filter was positioned between the beaker and the emitting lamp. The reactor was kept at room temperature (24 ± 3 °C) using a fan placed inside the reactor positioned a few centimeters aside from the beaker. The reaction mixtures were stirred using a magnetic stirrer bar of equal length for 30 min in the dark before irradiation to attain the adsorption–desorption equilibrium of the substrate. The reactions were stopped after 60 min. The suspensions were centrifuged to separate the catalysts from the dye solution and analyzed by UV-vis spectroscopy, considering the main absorption peak of this dye in the visible range, located at 554 nm. To ensure the evaluation of the photocatalytic activity measurements, tests of the RhB dye were carried out under visible light irradiation in the absence of the catalyst. The photocatalytic reactions were repeated three times to ensure the reproducibility of the data. The degradation % is calculated using Formula (1).

## 4. Conclusions

In summary, undoped ZnO and Al-doped ZnO quasi-spherical nanoparticles were successfully synthesized using a very accessible method and tested under UV and green light irradiation for the photodegradation of Rhodamine B in an aqueous solution. The ICP-OES analysis confirmed the successful incorporation of the dopant into the final solid. X-ray diffraction showed that all the samples exhibit the hexagonal wurtzite structure. After incorporating the aluminum into the zinc oxide lattice, additional peaks appeared, which correspond to the secondary phase of Zn_6_Al_2_(OH)_16_CO_3_∙4H_2_O. The TEM images displayed that the quasi-spherical nanoparticles grow with the insertion of the dopant and, due to the annealing temperature, tended to agglomerate. The ^27^Al NMR elucidated the position occupied by aluminum in the ZnO lattice, highlighting the migration from the octahedral coordination, which is the preferred site for the as-prepared materials, towards tetrahedral coordination after annealing in nitrogen flow. The FT-IR technique was sensitive to the variations of particle morphology and changes in the charge carriers pre- and post-annealing. PL allowed an in-depth study of the catalyst’s defects.

CZO, ZO and AZO catalysts were investigated as photocatalysts for degrading Rhodamine B dye in an aqueous solution under UV-visible light irradiation. Under UV-light irradiation, all the materials underperformed with respect to commercial zinc oxide. Undoped ZnO exhibited the best photocatalytic performance when illuminated under green light (525 nm), as demonstrated by the high degree of discoloration with respect to the commercial ZnO evaluated under the same conditions. The insertion of the dopant resulted in lower photocatalytic activity under UV-light irradiation and dropped the efficiency of the AZO catalysts to zero under green light irradiation. The catalysts were also thermally treated under a nitrogen atmosphere at 400 and 550 °C and evaluated again for the discoloration of the dye. Results of the present work demonstrated that annealing undoped zinc oxide nanoparticles at 400 °C for 10 min under nitrogen flow improves the discoloration efficiency by up to 81%. These results strongly suggest the interconnection between defects, synthesis and post-synthesis route, particle size and photocatalytic activity. The photocatalytic performances under green light irradiation can be explained as follows: CZO mainly contains zinc interstitial and has emission centers in the violet–blue region that are inactive for the photodegradation of RhB dye under this excitation wavelength. Undoped ZO presents red-emitting centers, which can be associated with a high content of O_2_^2−^, which is the main initiator of the photodegradation and responsible for the discoloration of the dye [7,71]. Annealing ZO at 400 and 550 °C modified the morphology and the size distribution along with the aggregation. Compared to the ZO and ZO-400 samples, the degree of aggregation is larger in ZO-550. Therefore, the substance is paying the penalty of surface area reduction induced by the high temperature. The improved photocatalytic activity of ZO-400 might be ascribed to the different morphology displayed by the particles after annealing. The effect of aluminum doping was studied as well: even the insertion of a small percent of dopant in the zinc oxide lattice, such as in AZO-05, leads to a material with a significant number of oxygen vacancies (green band, PL). As shown by the PL measured at 532 nm, there is not much difference in the spectra between ZO, AZO-05 and AZO-5. Here, we suggest that a small amount (equal or less than 0.5%) of aluminum occupying substitutional tetrahedral sites of the ZnO structure, saturates the photocatalytically active sites on the catalyst surface. Higher amounts of aluminum doping surpass the solubility limit and tend to occupy octahedral positions in secondary phases as well. As a consequence, oxygen vacancies are created in the ZnO, which act as fast-charge recombination centers. Moreover, the higher amount of this type of defect, that do not absorb in the green region, and a reduction of O_2_^2−^ species, possibly deactivate the photocatalytic process making the AZO catalysts inactive for the photodegradation of the RhB dye in an aqueous solution under green light.

## Figures and Tables

**Table 1:** Undoped, Al-doped and commercial ZnO sample codes.

Sample Type	Sample Code	Nominal Al Concentration (at%)	Annealing in N_2_ Flow
Commercial ZnO	**CZO**	0	/
Undoped ZnO	**ZO**	0	/
Undoped ZnO	**ZO-400**	0	400 °C/10 min
Undoped ZnO	**ZO-550**	0	550 °C/10 min
Al-doped ZnO	**AZO-05**	0.5	/
Al-doped ZnO	**AZO-05-400**	0.5	400 °C/10 min
Al-doped ZnO	**AZO-05-550**	0.5	550 °C/10 min
Al-doped ZnO	**AZO-1**	1	/
Al-doped ZnO	**AZO-2**	2	/
Al-doped ZnO	**AZO-3**	3	/
Al-doped ZnO	**AZO-5**	5	/
Al-doped ZnO	**AZO-5-400**	5	400 °C/10 min
Al-doped ZnO	**AZO-5-550**	5	550 °C/10 min

**Table 2:** Degradation of RhB after 20 min of UV-C and UV-A light irradiation in the presence of commercial ZnO, undoped ZnO, 0.5 and 5 at% Al-doped ZnO nanopowders.

Sample Codes	RhB Dye Degradation [%] UV-C 254 nm	RhB Dye Degradation [%] UV-A 368 nm
CZO	94 ± 1	95.9 ± 0.1
ZO	44.6 ± 0.5	70 ± 3
AZO-05	41 ± 2	53 ± 2
AZO-5	21 ± 2	19 ± 3

**Table 3:** Degradation of RhB under green light irradiation for one hour and four hours in the presence of CZO, ZO, AZO-05 and AZO-5 nanomaterials before and after the thermal treatment under nitrogen at 400 and 550 °C.

RhB Dye Degradation (%) under Green Light (525 nm)				
Sample Codes	Reaction Time	Not Annealed	400 °C in N_2_	550 °C in N_2_
CZO	1 h	20 ± 4	20 ± 4	19 ± 2
ZO	1 h	39 ± 1	50 ± 3	21 ± 1
AZO-05	1 h	26 ± 2	21.7 ± 0.5	25 ± 1
AZO-5	1 h	21 ± 2	22 ± 1	23 ± 1
ZO	4 h	64 ± 2	81 ± 4	51.3 ± 0.5

## Data Availability

Not applicable.

## Supplementary Materials

The following supporting information can be downloaded at: https://www.mdpi.com/article/10.3390/ijms232415459/s1.

## References

[1] UNESCO (2020). The United Nations World Water Development Report 2020_ Water and Climate Change.

[2] Lim H., Yusuf M., Song S., Park S., Park K.H. (2021). Efficient Photocatalytic Degradation of Dyes Using Photo-Deposited Ag Nanoparticles on ZnO Structures: Simple Morphological Control of ZnO. RSC Adv..

[3] Mahapatra N.N. (2016). Textile Dyes.

[4] Thomas Bechtold T.P. (2019). Textile Chemistry.

[5] Reddy P.A.K., Reddy P.V.L., Kwon E., Kim K.-H., Akter T., Kalagara S. (2016). Recent Advances in Photocatalytic Treatment of Pollutants in Aqueous Media. Environ. Int..

[6] Balcha A., Yadav O.P., Dey T. (2016). Photocatalytic Degradation of Methylene Blue Dye by Zinc Oxide Nanoparticles Obtained from Precipitation and Sol-Gel Methods. Environ. Sci. Pollut. Res..

[7] Dodoo-Arhin D., Asiedu T., Agyei-Tuffour B., Nyankson E., Obada D., Mwabora J.M. (2021). Photocatalytic Degradation of Rhodamine Dyes Using Zinc Oxide Nanoparticles. Mater. Today Proc..

[8] Ahlström L.-H., Sparr Eskilsson C., Björklund E. (2005). Determination of Banned Azo Dyes in Consumer Goods. TrAC Trends Anal. Chem..

[9] Khan S., Malik A. (2018). Toxicity Evaluation of Textile Effluents and Role of Native Soil Bacterium in Biodegradation of a Textile Dye. Environ. Sci. Pollut. Res..

[10] Sansenya T., Masri N., Chankhanittha T., Senasu T., Piriyanon J., Mukdasai S., Nanan S. (2022). Hydrothermal Synthesis of ZnO Photocatalyst for Detoxification of Anionic Azo Dyes and Antibiotic. J. Phys. Chem. Solids.

[11] Khataee A.R., Pons M.N., Zahraa O. (2009). Photocatalytic Degradation of Three Azo Dyes Using Immobilized TiO2 Nanoparticles on Glass Plates Activated by UV Light Irradiation: Influence of Dye Molecular Structure. J. Hazard. Mater..

[12] John Peter I., Praveen E., Vignesh G., Nithiananthi P. (2017). ZnO Nanostructures with Different Morphology for Enhanced Photocatalytic Activity. Mater. Res. Express.

[13] Ani I.J., Akpan U.G., Olutoye M.A., Hameed B.H. (2018). Photocatalytic Degradation of Pollutants in Petroleum Refinery Wastewater by TiO2- and ZnO-Based Photocatalysts: Recent Development. J. Clean. Prod..

[14] Sinar Mashuri S.I., Ibrahim M.L., Kasim M.F., Mastuli M.S., Rashid U., Abdullah A.H., Islam A., Asikin Mijan N., Tan Y.H., Mansir N. (2020). Photocatalysis for Organic Wastewater Treatment: From the Basis to Current Challenges for Society. Catalysts.

[15] Natarajan T.S., Thomas M., Natarajan K., Bajaj H.C., Tayade R.J. (2011). Study on UV-LED/TiO2 Process for Degradation of Rhodamine B Dye. Chem. Eng. J..

[16] Ayaz S., Amin R., Samantray K., Dasgupta A., Sen S. (2021). Tunable Ultraviolet Sensing Performance of Al-Modified ZnO Nanoparticles. J. Alloys Compd..

[17] Saber O., El-Brolossy T.A., Al Jaafari A.A. (2012). Improvement of Photocatalytic Degradation of Naphthol Green B Under Solar Light Using Aluminum Doping of Zinc Oxide Nanoparticles. Water Air Soil Pollut..

[18] Wu J., Xue D. (2011). Progress of Science and Technology of ZnO as Advanced Material. Sci. Adv. Mater..

[19] Momot A., Amini M.N., Reekmans G., Lamoen D., Partoens B., Slocombe D.R., Elen K., Adriaensens P., Hardy A., Van Bael M.K. (2017). A Novel Explanation for the Increased Conductivity in Annealed Al-Doped ZnO: An Insight into Migration of Aluminum and Displacement of Zinc. Phys. Chem. Chem. Phys..

[20] Buonsanti R., Llordes A., Aloni S., Helms B.A., Milliron D.J. (2011). Tunable Infrared Absorption and Visible Transparency of Colloidal Aluminum-Doped Zinc Oxide Nanocrystals. Nano Lett..

[21] Della Gaspera E., Chesman A.S.R., van Embden J., Jasieniak J.J. (2014). Non-Injection Synthesis of Doped Zinc Oxide Plasmonic Nanocrystals. ACS Nano.

[22] Garcia G., Buonsanti R., Runnerstrom E.L., Mendelsberg R.J., Llordes A., Anders A., Richardson T.J., Milliron D.J. (2011). Dynamically Modulating the Surface Plasmon Resonance of Doped Semiconductor Nanocrystals. Nano Lett..

[23] Jaramillo-Páez C., Sánchez-Cid P., Navío J.A., Hidalgo M.C. (2018). A Comparative Assessment of the UV-Photocatalytic Activities of ZnO Synthesized by Different Routes. J. Environ. Chem. Eng..

[24] Khalid N.R., Hammad A., Tahir M.B., Rafique M., Iqbal T., Nabi G., Hussain M.K. (2019). Enhanced Photocatalytic Activity of Al and Fe Co-Doped ZnO Nanorods for Methylene Blue Degradation. Ceram. Int..

[25] Bazzani M., Neroni A., Calzolari A., Catellani A. (2011). Optoelectronic Properties of Al:ZnO: Critical Dosage for an Optimal Transparent Conductive Oxide. Appl. Phys. Lett..

[26] Abrinaei F., Molahasani N. (2018). Effects of Mn Doping on the Structural, Linear, and Nonlinear Optical Properties of ZnO Nanoparticles. J. Opt. Soc. Am. B.

[27] Calzolari A., Catellani A. (2017). Doping, Co-Doping, and Defect Effects on the Plasmonic Activity of ZnO-Based Transparent Conductive Oxides. Oxide-Based Mater. Devices VIII.

[28] Yaqoob A.A., Mohd Noor N.H.b., Serrà A., Mohamad Ibrahim M.N. (2020). Advances and Challenges in Developing Efficient Graphene Oxide-Based ZnO Photocatalysts for Dye Photo-Oxidation. Nanomaterials.

[29] Damm H., Adriaensens P., De Dobbelaere C., Capon B., Elen K., Drijkoningen J., Conings B., Manca J.V., D’Haen J., Detavernier C. (2014). Factors Influencing the Conductivity of Aqueous Sol(Ution)–Gel-Processed Al-Doped ZnO Films. Chem. Mater..

[30] Kelchtermans A., Elen K., Schellens K., Conings B., Damm H., Boyen H.G., D’Haen J., Adriaensens P., Hardy A., Van Bael M.K. (2013). Relation between Synthesis Conditions, Dopant Position and Charge Carriers in Aluminium-Doped ZnO Nanoparticles. RSC Adv..

[31] Serier H., Gaudon M., Ménétrier M. (2009). Al-Doped ZnO Powdered Materials: Al Solubility Limit and IR Absorption Properties. Solid State Sci..

[32] Conversion B., Pant K.K., Gupta S.K., Ahmad E. (2021). Catalysis for Clean Energy and Environmental Sustainability.

[33] Nandi P., Das D. (2019). Photocatalytic Degradation of Rhodamine-B Dye by Stable ZnO Nanostructures with Different Calcination Temperature Induced Defects. Appl. Surf. Sci..

[34] Munawar T., Yasmeen S., Hussain F., Mahmood K., Hussain A., Asghar M., Iqbal F. (2020). Synthesis of Novel Heterostructured ZnO-CdO-CuO Nanocomposite: Characterization and Enhanced Sunlight Driven Photocatalytic Activity. Mater. Chem. Phys..

[35] Neena D., Kondamareddy K.K., Bin H., Lu D., Kumar P., Dwivedi R.K., Pelenovich V.O., Zhao X.-Z., Gao W., Fu D. (2018). Enhanced Visible Light Photodegradation Activity of RhB/MB from Aqueous Solution Using Nanosized Novel Fe-Cd Co-Modified ZnO. Sci. Rep..

[36] Burdett J.K., Price G.D., Price S.L. (1982). Role of the Crystal-Field Theory in Determining the Structures of Spinels. J. Am. Chem. Soc..

[37] Kelchtermans A., Adriaensens P., Slocombe D., Kuznetsov V.L., Hadermann J., Riskin A., Elen K., Edwards P.P., Hardy A., Van Bael M.K. (2015). Increasing the Solubility Limit for Tetrahedral Aluminium in ZnO:Al Nanorods by Variation in Synthesis Parameters. J. Nanomater..

[38] Tsubota T., Ohtaki M., Eguchi K., Arai H. (1997). Thermoelectric Properties of Al-Doped ZnO as a Promising Oxide Material for High-Temperature Thermoelectric Conversion. J. Mater. Chem..

[39] Wan Shick H., De Jonghe L.C., Xi Y., Rahaman M.N. (1995). Reaction Sintering of ZnO-Al2O3. J. Am. Ceram. Soc..

[40] Thu T.V., Maenosono S. (2010). Synthesis of High-Quality Al-Doped ZnO Nanoink. J. Appl. Phys..

[41] Serier H., Demourgues A., Majimel J., Gaudon M. (2011). Infrared Absorptive Properties of Al-Doped ZnO Divided Powder. J. Solid State Chem..

[42] Suwanboon S., Amornpitoksuk P., Haidoux A., Tedenac J.C. (2008). Structural and Optical Properties of Undoped and Aluminium Doped Zinc Oxide Nanoparticles via Precipitation Method at Low Temperature. J. Alloys Compd..

[43] Kumar R.S., Sathyamoorthy R., Sudhagar P., Matheswaran P., Hrudhya C.P., Kang Y.S. (2011). Effect of Aluminum Doping on the Structural and Luminescent Properties of ZnO Nanoparticles Synthesized by Wet Chemical Method. Phys. E Low-Dimens. Syst. Nanostruct..

[44] Hartner S., Ali M., Schulz C., Winterer M., Wiggers H. (2009). Electrical Properties of Aluminum-Doped Zinc Oxide (AZO) Nanoparticles Synthesized by Chemical Vapor Synthesis. Nanotechnology.

[45] Brehm J.U., Winterer M., Hahn H. (2006). Synthesis and Local Structure of Doped Nanocrystalline Zinc Oxides. J. Appl. Phys..

[46] Kelchtermans A. (2014). Synthesis and In-Depth Characterization of Al-Doped ZnO Nanoparticles as Building Blocks for TCO Layers. Ph.D. Thesis.

[47] Pinna N., Garnweitner G., Antonietti M., Niederberger M. (2005). A General Nonaqueous Route to Binary Metal Oxide Nanocrystals Involving a C−C Bond Cleavage. J. Am. Chem. Soc..

[48] Avadhut Y.S., Weber J., Hammarberg E., Feldmann C., Schmedtaufder Günne J. (2012). Structural Investigation of Aluminium Doped ZnO Nanoparticles by Solid-State NMR Spectroscopy. Phys. Chem. Chem. Phys..

[49] Titova Y.Y., Schmidt F.K. (2022). What 27Al NMR Spectroscopy Can Offer to Study of Multicomponent Catalytic Hydrogenation Systems?. J. Organomet. Chem..

[50] Roberts N., Wang R., Sleight A.W. (1998). And Impurity Nuclear Magnetic Resonance in ZnO:Al and ZnO:Ga. Phys. Rev. B Condens. Matter Mater. Phys..

[51] Knight W.D. (1949). Nuclear Magnetic Resonance Shift in Metals. Phys. Rev..

[52] Achehboune M., Khenfouch M., Boukhoubza I., Leontie L., Doroftei C., Carlescu A., Bulai G., Mothudi B., Zorkani I., Jorio A. (2022). Microstructural, FTIR and Raman Spectroscopic Study of Rare Earth Doped ZnO Nanostructures. Mater. Today Proc..

[53] Djelloul A., Aida M.S., Bougdira J. (2010). Photoluminescence, FTIR and X-ray Diffraction Studies on Undoped and Al-Doped ZnO Thin Films Grown on Polycrystalline α-Alumina Substrates by Ultrasonic Spray Pyrolysis. J. Lumin..

[54] Mojumder S., Das T., Das S., Chakraborty N., Saha D., Pal M. (2022). Y and Al Co-Doped ZnO-Nanopowder Based Ultrasensitive Trace Ethanol Sensor: A Potential Breath Analyzer for Fatty Liver Disease and Drunken Driving Detection. Sens. Actuators B Chem..

[55] Zhang X., Chen Y., Zhang S., Qiu C. (2017). High Photocatalytic Performance of High Concentration Al-Doped ZnO Nanoparticles. Sep. Purif. Technol..

[56] Munawaroh H., Wahyuningsih S., Ramelan A.H. (2017). Synthesis and Characterization of Al Doped ZnO (AZO) by Sol-Gel Method. IOP Conf. Ser. Mater. Sci. Eng..

[57] Sowri Babu K., Ramachandra Reddy A., Sujatha C., Venugopal Reddy K., Mallika A.N. (2013). Synthesis and Optical Characterization of Porous ZnO. J. Adv. Ceram..

[58] Mihai G.D., Meynen V., Mertens M., Bilba N., Cool P., Vansant E.F. (2010). ZnO Nanoparticles Supported on Mesoporous MCM-41 and SBA-15: A Comparative Physicochemical and Photocatalytic Study. J. Mater. Sci..

[59] Liao Y., Xie C., Liu Y., Chen H., Li H., Wu J. (2012). Comparison on Photocatalytic Degradation of Gaseous Formaldehyde by TiO_2_, ZnO and Their Composite. Ceram. Int..

[60] Collard X., El Hajj M., Su B.-L., Aprile C. (2014). Synthesis of Novel Mesoporous ZnO/SiO2 Composites for the Photodegradation of Organic Dyes. Microporous Mesoporous Mater..

[61] Ajala F., Hamrouni A., Houas A., Lachheb H., Megna B., Palmisano L., Parrino F. (2018). The Influence of Al Doping on the Photocatalytic Activity of Nanostructured ZnO: The Role of Adsorbed Water. Appl. Surf. Sci..

[62] Hamrouni A., Moussa N., Parrino F., Di Paola A., Houas A., Palmisano L. (2014). Sol-Gel Synthesis and Photocatalytic Activity of ZnO-SnO_2_ Nanocomposites. J. Mol. Catal. A Chem..

[63] Amalia F.R., Takashima M., Ohtani B. (2022). Are You Still Using Organic Dyes? Colorimetric Formaldehyde Analysis for True Photocatalytic-Activity Evaluation. Chem. Commun..

[64] Schuster A. (1905). Radiation Through a Foggy Atmosphere. Astrophys. J..

[65] Kubelka P., Munk F., KUBELKA P. (1931). Ein Beitrag Zur Optik Der Farbanstriche. Z. Tech. Phys..

[66] Kubelka P. (1948). New Contributions to the Optics of Intensely Light-Scattering Materials Part I. J. Opt. Soc. Am..

[67] Kubelka P. (1954). New Contributions to the Optics of Intensely Light-Scattering Materials Part II: Nonhomogeneous Layers. J. Opt. Soc. Am..

[68] Zanatta A.R. (2019). Revisiting the Optical Bandgap of Semiconductors and the Proposal of a Unified Methodology to Its Determination. Sci. Rep..

[69] Paramo J.A., Strzhemechny Y.M., Endo T., Crnjak Orel Z. (2015). Correlation of Defect-Related Optoelectronic Properties in Zn_5_(OH)_6_(CO_3_)_2_/ZnO Nanostructures with Their Quasi-Fractal Dimensionality. J. Nanomater..

[70] Zhang M., Averseng F., Haque F., Borghetti P., Krafft J.M., Baptiste B., Costentin G., Stankic S. (2019). Defect-Related Multicolour Emissions in ZnO Smoke: From Violet, over Green to Yellow. Nanoscale.

[71] Pal M., Bera S., Sarkar S., Jana S. (2014). Influence of Al Doping on Microstructural, Optical and Photocatalytic Properties of Sol–Gel Based Nanostructured Zinc Oxide Films on Glass. RSC Adv..

