# Supplementary material for: Photocatalytic Performance of Undoped and Al-Doped ZnO Nanoparticles in the Degradation of Rhodamine B under UV-Visible Light:The Role of Defects and Morphology
Source: Int J Mol Sci. 2022 Dec 7;23(24):15459. doi: 10.3390/ijms232415459 (PMC9779551; doi:10.3390/ijms232415459)
Supplement: Supplementary file 1 [file ijms-23-15459-s001.zip › ijms-2050613-SI.pdf]

SUPPLEMENTARY INFORMATION

Photocatalytic Performance of Undoped and Al-doped ZnO Nanoparticles in the Degradation of Rhodamine B under UV-Visible Light: The Role of Defects and Morphology

Alessandra Piras[1,2], Chiara Olla[3], Gunter Reekmans[4], An-Sofie Kelchtermans[2,5], Dries De Sloovere[2,5,6], Ken Elen[2,5,6], Carlo Maria Carbonaro[3], Luca Fusaro[1], Peter Adriaensens[4], An Hardy[2,5,6], Carmela Aprile[1], Marlies K. Van Bael[2,5,6*]

1 University of Namur, Laboratory of Applied Materials Chemistry, Unit of Nanomaterials Chemistry, Chemistry Department, NISM, Rue de Bruxelles, 61, 5000 Namur, Belgium
2 Hasselt University, Institute for Materials Research (imo-imomec), DESINe Group, Agoralaan building D, 3590 Diepenbeek, Belgium
3 University of Cagliari, Department of Physics, Cittadella Universitaria, I-09042 Monserrato, Italy
4 Hasselt University, Institute for Materials Research (imo-imomec), Analytical and Circular Chemistry (ACC), Agoralaan building D, 3590 Diepenbeek, Belgium
5 EnergyVille, Thor Park 8320, 3600 Genk, Belgium
6 Imec, Division Imomec, Wetenschapspark 1, 3590 Diepenbeek, Belgium
* Correspondence: marlies.vanbael@uhasselt.be

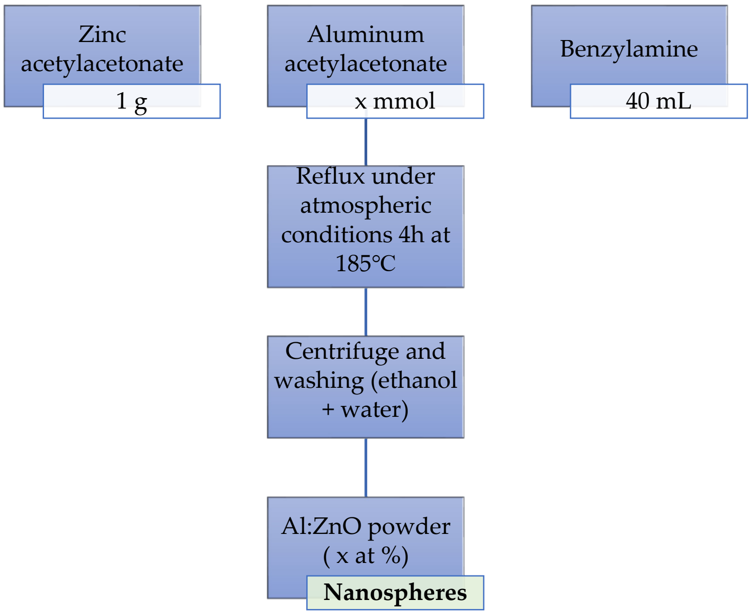

Figure S1. Synthesis scheme for AZO quasi-spherical nanoparticles.

Table S1. Nominal and actual % of aluminum content into the as-synthesized Al-doped ZnO solids.

Sample codes	Nominal Al content	Actual Al content
AZO-05	0.05	0.42
AZO-1	1	0.89
AZO-2	2	1.81
AZO-3	3	2.66
AZO-5	5	3.79

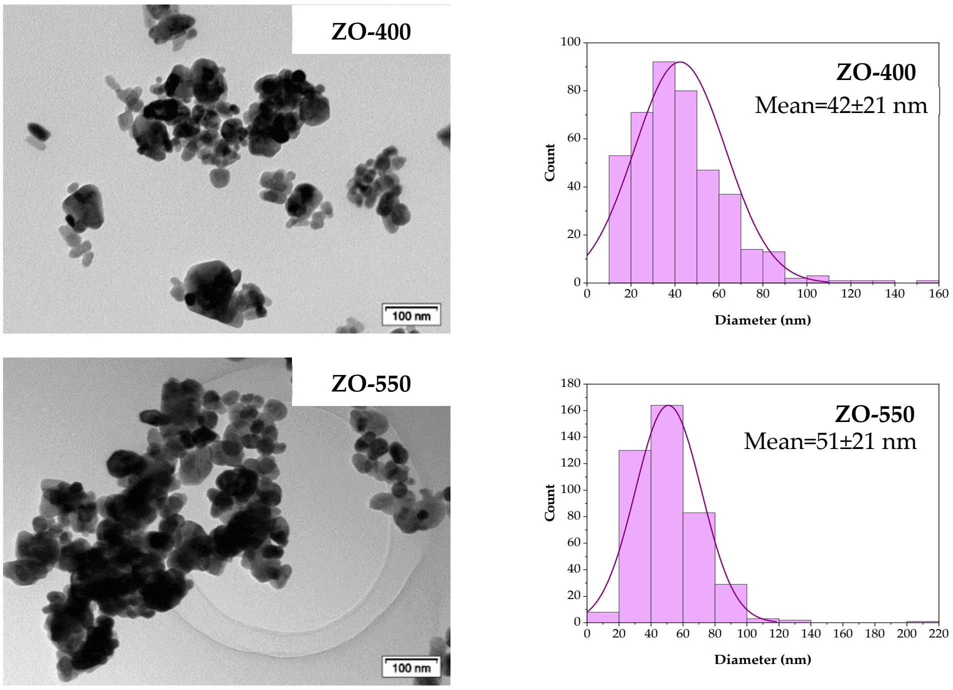

Figure S2. TEM micrographs of ZnO annealed at 400 and 550 °C in N_2 atmosphere supported with the corresponding particle size histograms.

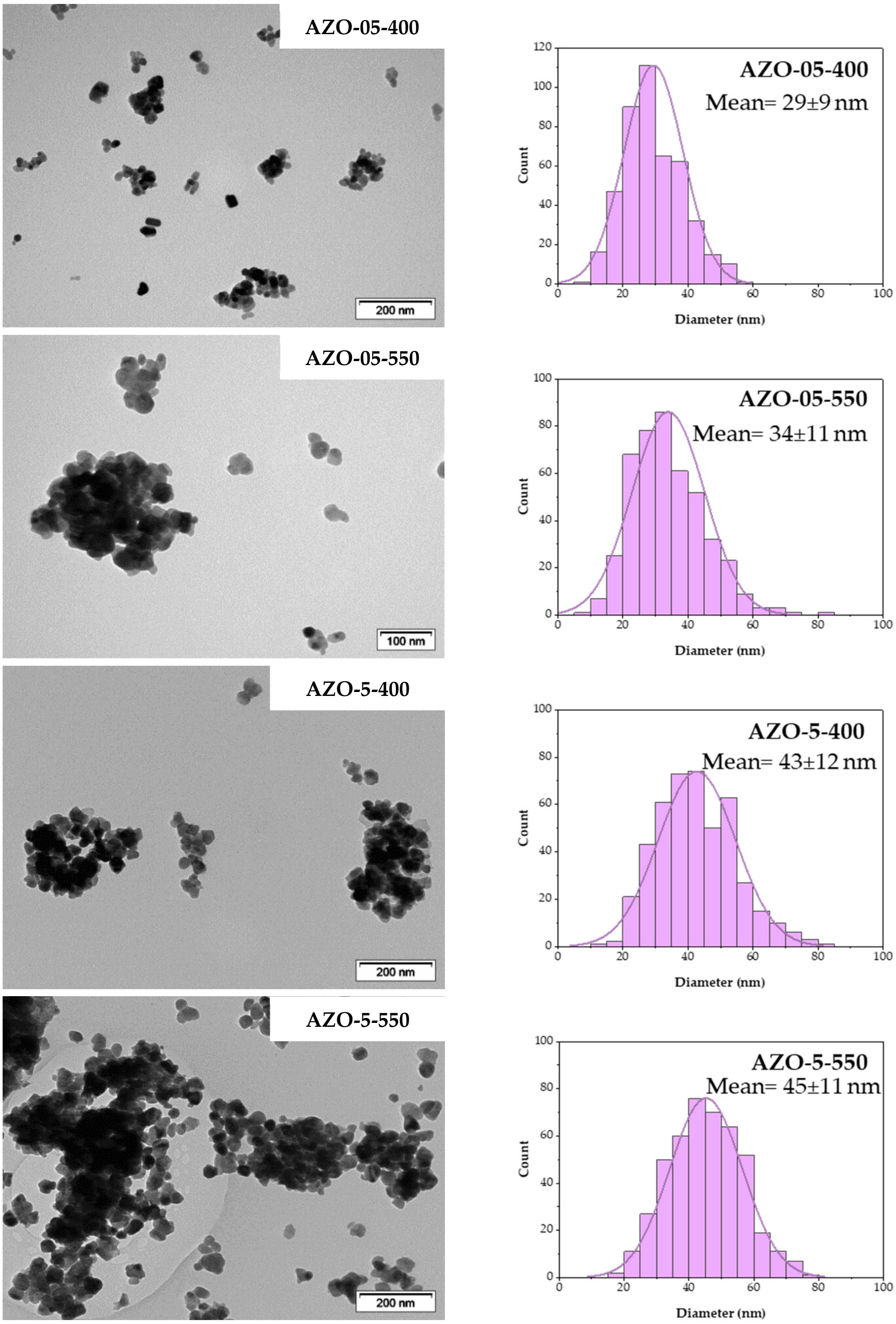

Figure S3. TEM micrographs of the AZOs annealed at 400 and 550 °C in N_2 atmosphere supported with the corresponding particle size histograms.

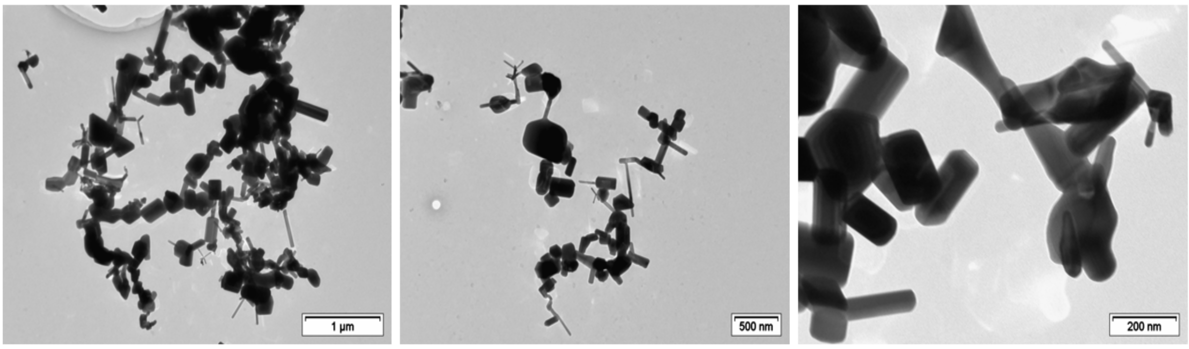

Figure S4. TEM micrographs of the commercial ZnO, CZO.

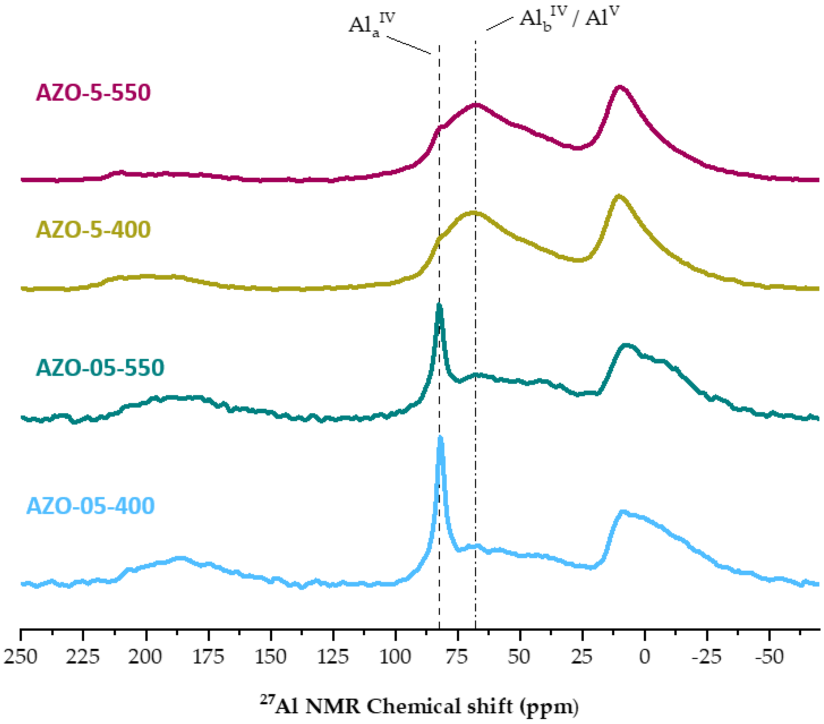

Figure S5. ^{27}Al MAS NMR comparison between AZO-05 and AZO-5 quasi-spherical nanoparticles annealed in N_2 at 400 and 550 °C. The two lines highlight Al_a^{IV}, assigned to four-fold coordinated aluminum and Al_b^{IV} / Al^V, assigned to distorted four-fold coordinated and five-fold coordinated aluminum sites, respectively.

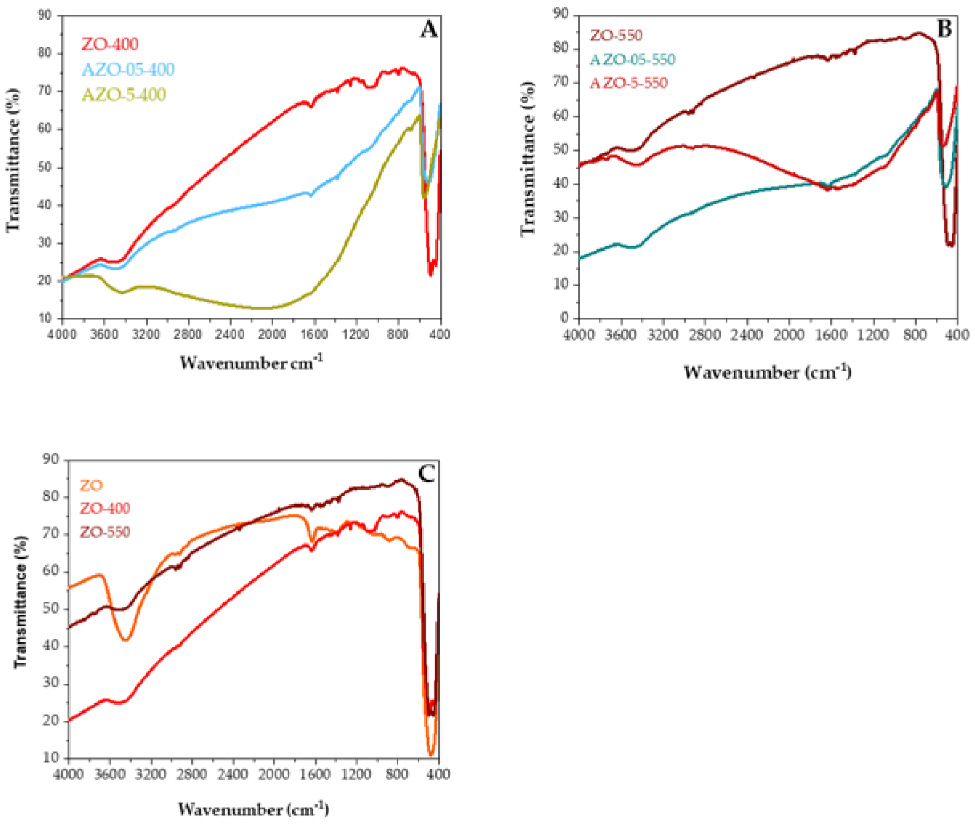

Figure S6. FT-IR spectra of the KBr pellets of quasi-spherical nanoparticles: A, comparison of ZO, AZO-05 and AZO-5 annealed at 400°C; B, comparison of ZO, AZO-05 and AZO-5 annealed at 550 °C; C, comparison of ZO as-synthesized and annealed at 400 and 550 °C.

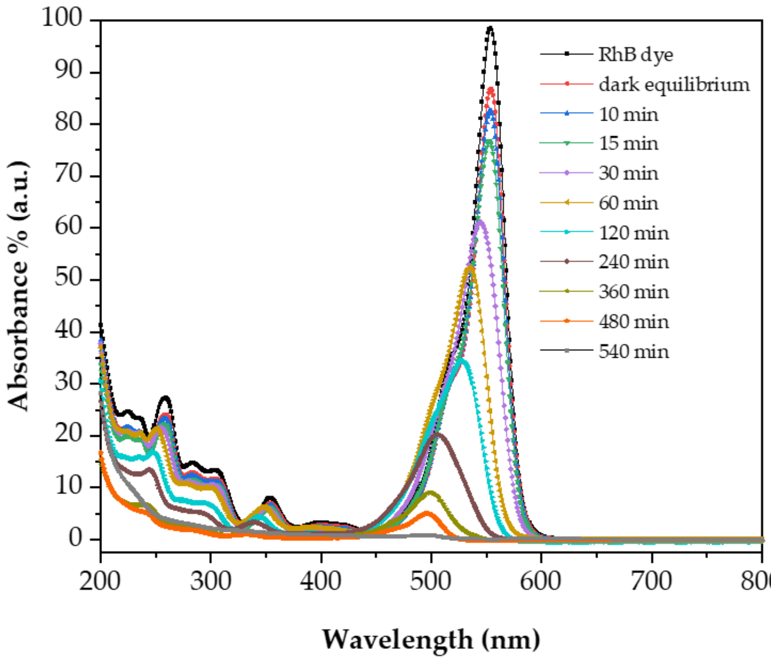

Figure S7. UV-Vis absorbance spectra of photodegraded Rhodamine B dye aqueous solutions with ZO-400 catalyst under green light irradiation at different time intervals. Rhodamine B dye adsorption during dark equilibrium is also reported.

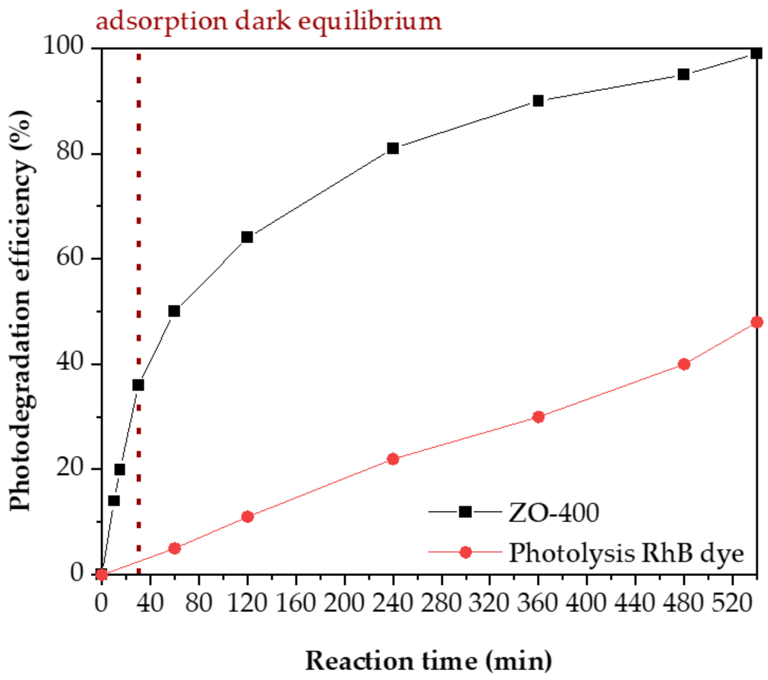

Figure S8. Photodegradation of Rhodamine B dye plotted as a function of the green light exposure time for the as-synthesized ZnO-400 material along with photolysis of Rhodamine B dye at indicated time intervals. The first 30 minutes correspond to RhB adsorption during dark equilibrium.

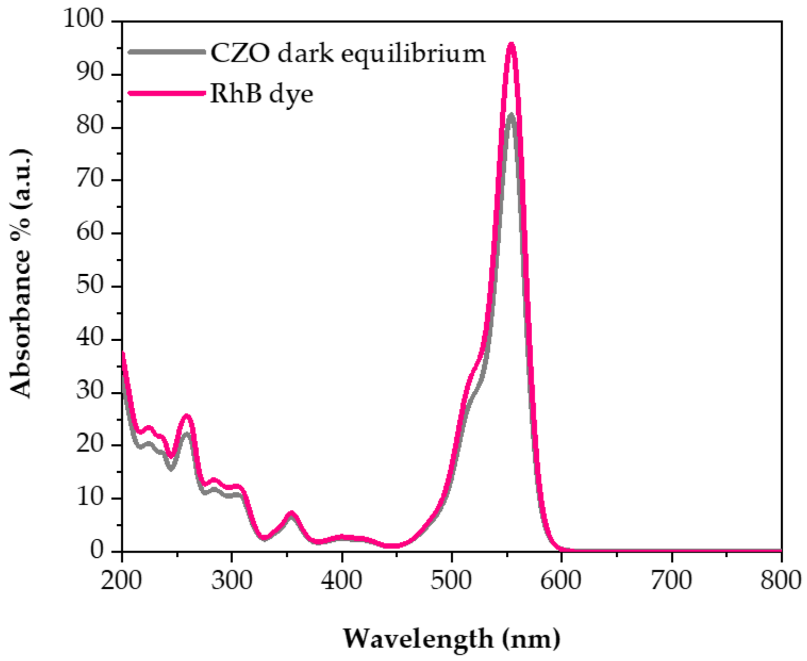

Figure S9. UV-Vis absorbance spectra of Rhodamine B dye without catalyst and Rhodamine B dye with CZO catalyst during one-hour dark equilibrium experiment.

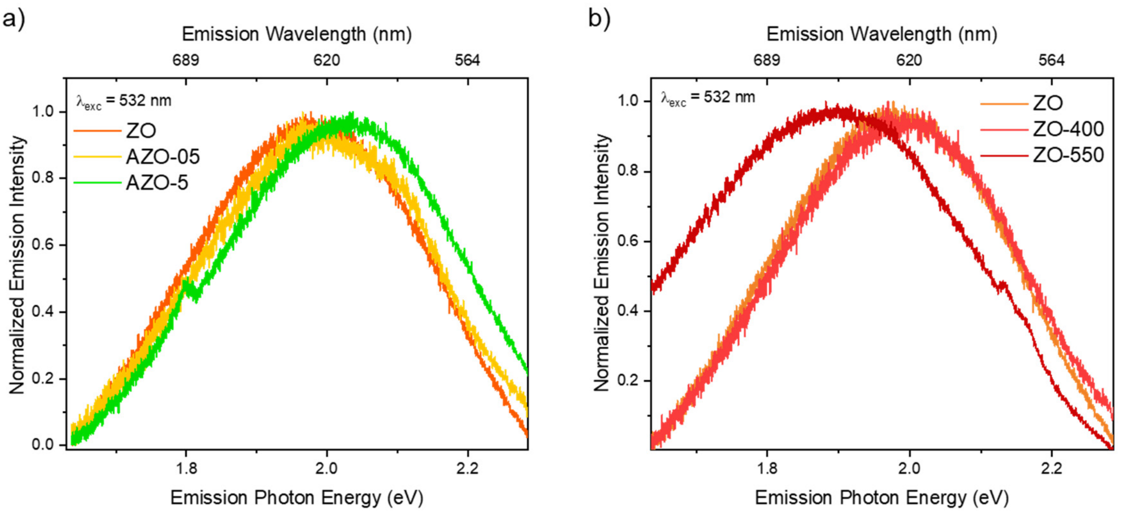

Figure S10. Normalized PL spectra of all samples excited at 532 nm. Comparison among different Al-doping percentages (b), and annealing temperatures (c) are displayed.

Table S2. Gaussian band deconvolution data of samples excited at 405 nm.

	Peak 1 (eV-nm)	Relative Area (%)	Peak 2 (eV-nm)	Relative Area (%)	Peak 3 (eV-nm)	Relative Area (%)
CZO	1.87 - 663	22	2.76 - 449	43	2.88 - 430	35
ZO	1.93 - 642	75	2.38 - 521	22	2.85 - 435	3
AZO-05	1.94 -639	71	2.39 - 519	26	2.86 - 433	3
AZO-5	2.06 - 602	47	2.44 - 508	53	-	-
ZO-400	1.94 -639	77	2.38 - 521	18	2.87 - 432	5
ZO-550	1.84 - 674	88	2.36 - 525	12	-	-

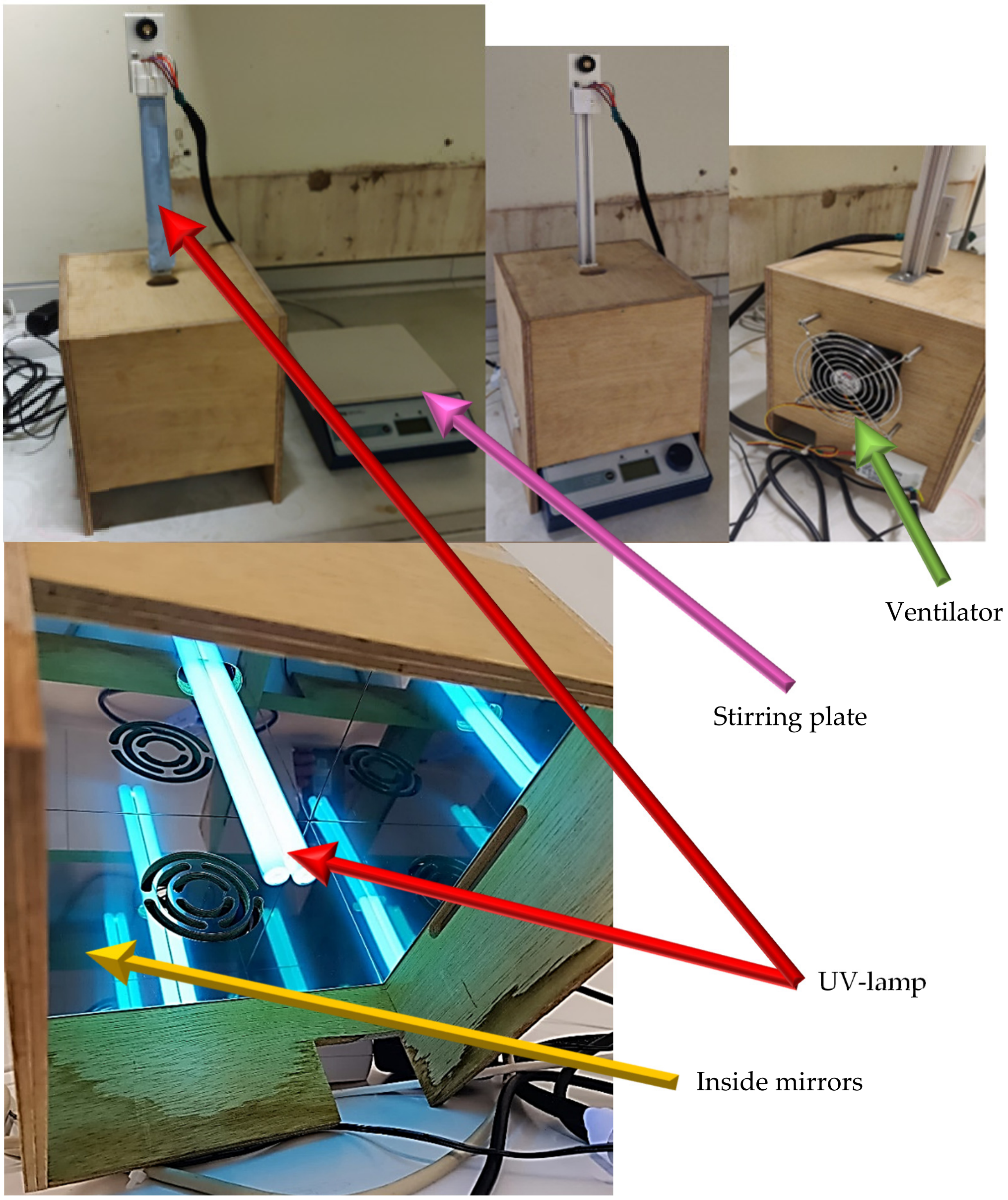

Figure S11. Home-made photoreactor. It consists of a wooden-box internally covered by mirrors, a pulley to carry the lamp, a lamp, a ballast, a socket, a magnetic stirrer plate with one single motor and four identical positions for beakers and a fan to keep the temperature constant while running reactions.

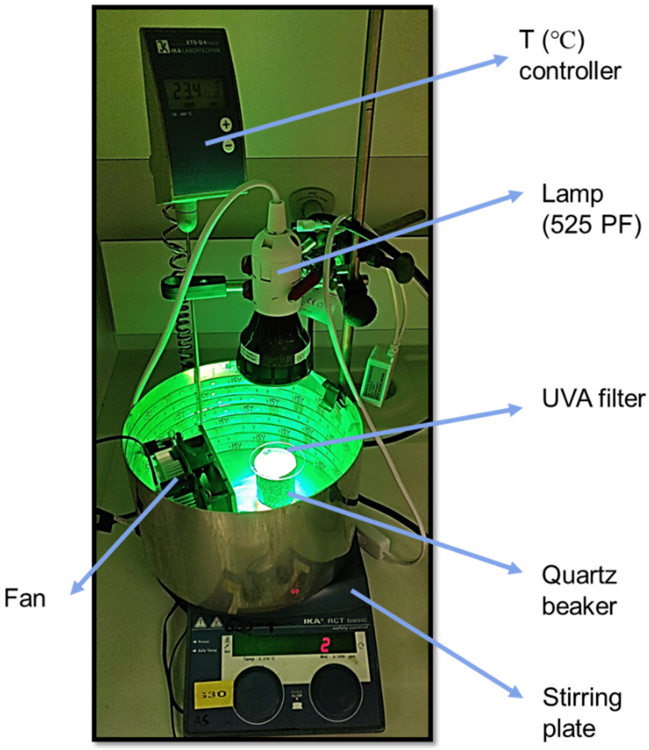

Figure S12. Home-made photoreactor working under visible light irradiation (green light, 525 nm). It consists of a metal-box, lamp support, a lamp, a ballast, a socket, a magnetic stirrer plate with one single motor with one position for a quartz beaker, temperature controller and a fan to keep the temperature constant while running reactions.

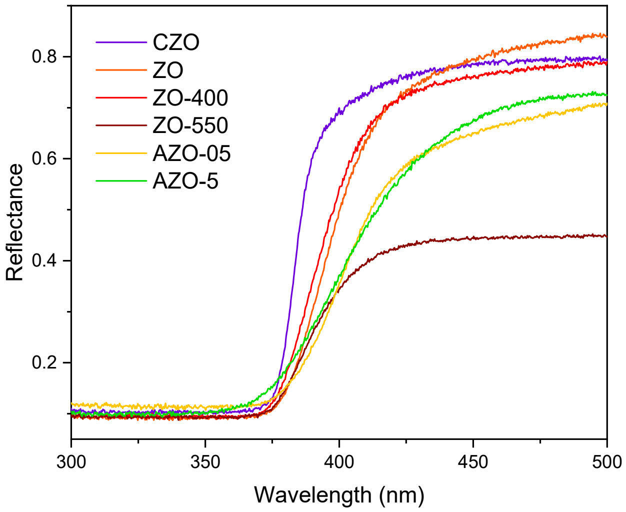

Figure S73. Reflectance spectra of the ZnO samples.

Calculation of the bandgap.

In order to calculate the bandgap of our powder samples, the reflectance values obtained were converted in a function F(R) of the apparent absorption K and diffuse reflection S coefficients via the Schuster-Kubelka-Munk formula[64-67]:

$$F(R_\infty) = \frac{(1 - R_\infty)^2}{2R_\infty} = \frac{K}{S}$$

Where R_∞ is the diffuse reflectance of the sample.
This pseudo-absorption function can be plotted as a function of energy and fitted with a sigmoid-Boltzmann function with good results according to a methodology recently studied by Zanatta in his excellent paper[68].

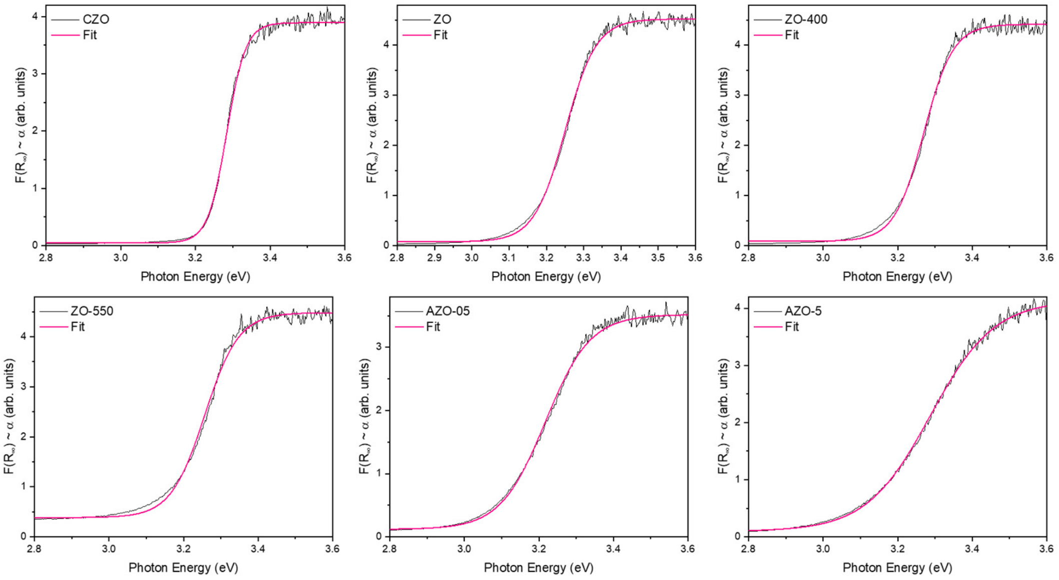

Figure S14. Plot of F(R) functions of our ZnO samples fitted with a sigmoidal-Boltzmann function.

Indeed, the fitting formula is:

$$\alpha(E) = \alpha_{max} + \frac{\alpha_{min} - \alpha_{max}}{1 + exp\left(\frac{E - E_0^{Boltz}}{\delta E}\right)}$$

Where α_{min} is the minimum absorption value, α_{max} is the maximum absorption value, E_0^{Boltz} is the energy value at which the absorption has an intermediate value between α_{min} and α_{max} and δE is the energy range linked to the slope of the curve.
Once fitted, the value of the bandgap E_{gap}^{Boltz} can be obtained according to this empirical relationship:

$$E_{gap}^{Boltz} = E_0^{Boltz} - n_{dir-ind}^{Boltz} \times \delta E$$

where $n_{dir-ind}^{Boltz}$ is a parameter experimentally determined that is equal to 0.3 for direct type bandgap (more details in Zanatta's work). In the following table, the calculated bandgaps.

Table S3. Calculated bandgaps.

	α_{min}	α_{max}	E_0^{Boltz} (eV)	δE (eV)	E_{gap}^{Boltz} (eV)	E_{gap}^{Boltz} (nm)
**CZO**	0.05	3.89	3.28	0.03	3.28	378
**ZO**	0.08	4.52	3.25	0.04	3.24	383
**ZO-400**	0.09	4.42	3.27	0.04	3.26	381
**ZO-550**	0.38	4.48	3.26	0.05	3.24	382
**AZO-05**	0.12	3.52	3.22	0.06	3.20	388
**AZO-5**	0.10	4.14	3.29	0.09	3.26	380
